# The Lethal Symbiont: Exploring the Pathophysiology of Cancer

**DOI:** 10.1152/physrev.00019.2025

**Published:** 2026-03-12

**Authors:** Emma Nolan, Leanne Li, Evangelos Giampazolias, Luigi Ombrato, Ilaria Malanchi

**Affiliations:** 1 Auckland Cancer Society Research Centre, University of Auckland, New Zealand; 2 Cancer-Neuroscience Laboratory, The Francis Crick Institute, London, UK; 3 Cancer Immunosurveillance Group, Cancer Research UK Manchester Institute, The University of Manchester, UK; 4 Centre for Tumour Microenvironment, Barts Cancer Institute, Queen Mary University of London, London, UK; 5 Tumour-Host Interaction Laboratory, The Francis Crick Institute, London, UK

## Abstract

From its early genesis, cancer is integrated with the surrounding tissue. Its very existence depends on surrounding normal tissue cells engaging with cancer cells to create an alternative tissue environment. This emerging abnormal structure becomes connected with the host organism via blood, lymphatic vessels, and neural connections. Through those connections, the cancer mass communicates and perturbs the entire organism altering various aspects of the steady state body physiology.

At early, asymptomatic stages, the induced changes within distant organs that harbour the potential to facilitate the spread of cancer are termed “premetastatic niche”. Many processes involved with pre-metastatic changes hijack processes typical in other context such as development, injury, or infections, but their co-occurrence creates a new alternative physiology.

The cancer to body connections not only have important consequences for the efficacy of cancer therapy but enable cancer to evolve and adapt under the very pressure of those treatments. Furthermore, as cancer induced changes are closely related to other physiological challenges, extrinsic perturbations such as diet, injury, and other inflammatory events, have strong impact on the tumour disease.

As the disease progresses, the complex intersection of inflammatory, metabolic, regenerative changes creates an escalating cascade of events causing cancer related syndrome, such as cachexia, that threaten the homeostasis of the entire body and can, per se, be deadly. In this article we will review the recent advances in the understanding of cancer as systemic malady.

## Part 1 Cancer to body connection

### 1.1 Local symbiosis, the tumour microenvironment (TME)

The growing evidence of the pervasive presence of somatic mutations in various healthy tissues^1,2^ indicates that, while oncogenic drivers are essential for cancer formation, whether a tumour will develop is determined by the tissue context with inflammation representing a long-known risk factor^3^. Indeed, external insults like pollutants causing a low-level inflammatory response were recently shown to expedite cancer formation^4^. Therefore, even before cancer occurs, the microenvironment of pre-cancerous cells harbouring mutations in oncogenic drivers needs to actively be involved in the tumorigenic process. Once cancer initiation occurs a completely distinct tissue ecosystem is created, with distinctive cellular and extracellular components, constantly supporting their growth. This ecosystem is termed tumour microenvironment (TME) and is constantly evolving during cancer progression^5^ as well as in response to anti-cancer therapy^6^. For these reasons, strategies to target the TME are emerging as promising novel therapies, with immunotherapy leading the way^7^.

**Figure 1:**
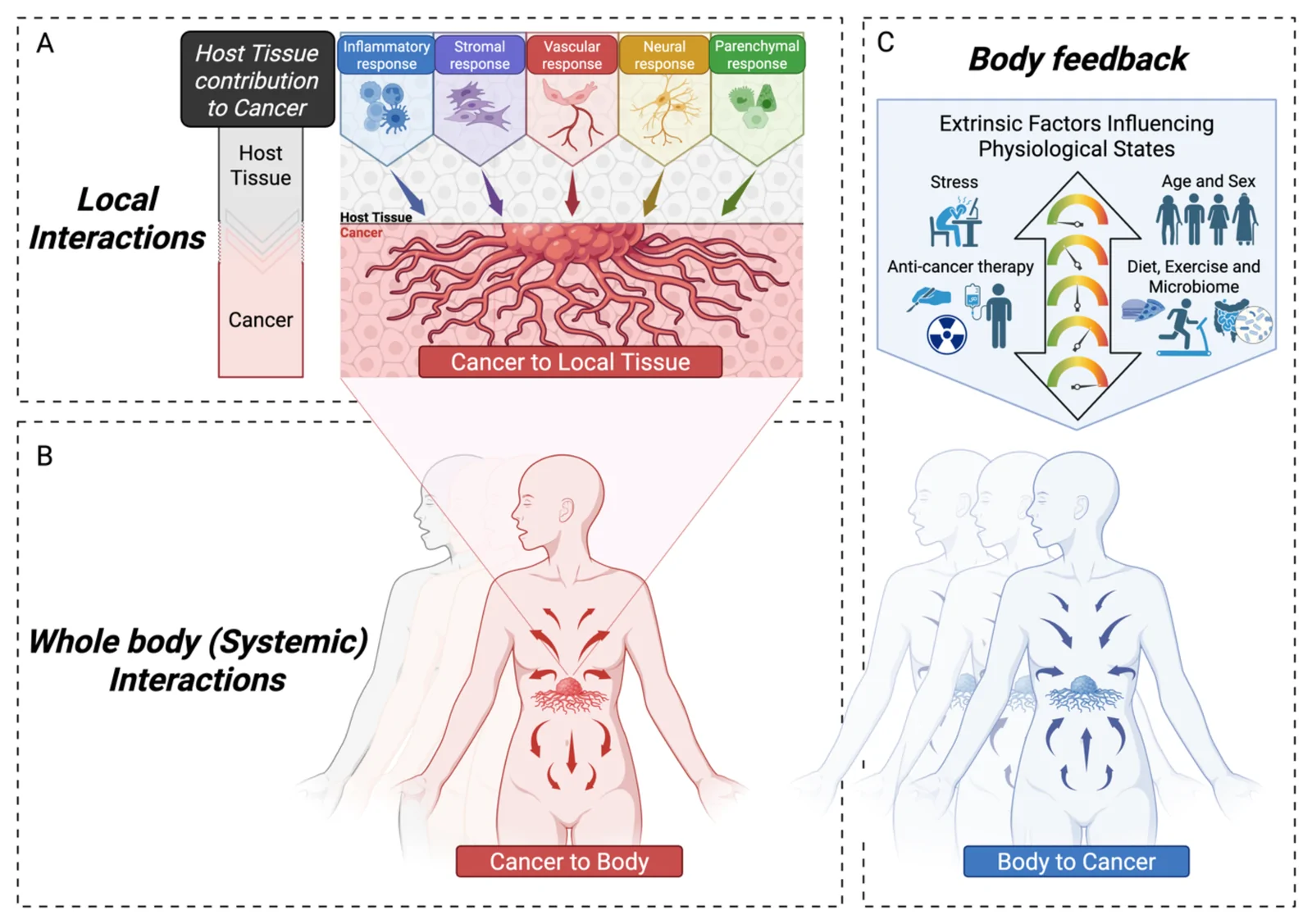
The complexity of the cancer-body connection. A and B represent the local (A) and the systemic whole-organism level perturbation (B) caused by a growing tumour discussed in the first part of this review under the *Cancer to Body connection*. Schematic representation of the key components within the local Tumour Micro-Environment (TME) resulting from the local interaction between cancer cells and the tissue of origin. C. Schematic representation of extrinsic factors altering the whole-body physiology and influencing the cancer disease discussed in the second part of this review within the *Body to Cancer connection*

In the next section, we will analyse the local symbiotic interactions between cancer cells and host tissue that leads to TME formation, with a focus on the major host cells constituting an essential part of the cancer structure (Figure 1A).

#### 1.1.1 Inflammatory response

Disruptions to tissue homeostasis—such as infection or injury—initiate an inflammatory response aimed at preserving tissue integrity. This involves the accumulation of leukocytes, cytokines, and plasma proteins at the affected site. Tissue-resident epithelial and immune cells detect damage via specialised receptors, triggering the production of pro-inflammatory mediators. These mediators drive vascular remodelling and recruit immune cells, ultimately leading to tissue repair and the restoration of homeostasis^8^. Depending on the timing, duration and the module of the inflammatory response, which is defined by the type and activation state of the immune cells and their associated cytokine and chemokine milieu, inflammation can exert an anti- or pro-tumorigenic function resulting in tumour control or outgrowth respectively^8^. However, if the cancer failed to be cleared during immune surveillance, inflammation will persist and pose strong evolutionary pressures that influence the onset and development of a primary tumour and eventually its metastatic potential^9^. Chronic inflammation has been associated with cancer development and is thought to contribute to approximately 15% of cancer lethality^8,10^. Only a small proportion of cancers cases are preceded by inflammation such as colitis-associated cancer, hepatitis-associated liver cancer and stomach cancer associated with *Helicobacter* infection^8,10^. More recently, elegant studies in the context of mouse model of pancreatitis have highlighted that the tissue inflammation co-operates with tissue cells harbouring the oncogenic driver Ras, diverting the normal process of tissue regeneration toward malignant transformation^11,12^. Moreover, Del Poggetto et al showed that after the resolution of acute inflammation, the tissue maintains an epithelial memory of the inflammatory response and this epigenetic priming predisposes for tumour initiation long after the tissue has recovered^13^. This epigenetic memory was shown to be reversible^14^, and highlights the complex relation between inflammation and tumour initiation.

Once tumours are initiated, inflammation regardless of its origin or the timing of its occurrence is an integral component of the TME. Inflammatory mediators, such as cytokines released by immune cells within the TME, can directly act as survival and growth factors to facilitate the proliferation and survival of transformed and malignant cells, ultimately driving tumour evolution^5^. Additionally, inflammation impacts the composition and function of other tumour stroma including fibroblasts and endothelial cells, remodelling the extracellular matrix to favour a niche for tumour expansion and increase cancer cell polarity and motility^8^. Collectively, cancer elicited inflammatory modules are highly plastic, displaying both immunostimulatory and immunosuppressive characteristics. Notably, environmental factors and nutrition such as obesity, smoking and pollution also contribute to cancer-related inflammation^4,15–17^. The dual nature of immune cell activation reflects the yin-yang of inflammation, which plays a pivotal role in regulating tumour initiation, progression, and metastasis. To fully unravel the mechanisms by which inflammation contributes to cancer, it is crucial to first understand how inflammation is initially triggered and sustained within the cancer context. This involves examining the timing of specific stimuli as well as the cellular and molecular participants that drive and perpetuate inflammatory processes in the TME.

##### Cancer-induced inflammation

During tumorigenesis transformed cells acquire a series of genetic and non-genetic molecular alterations that distinguishes them from healthy cells. The cancer-associated alterations can be picked up by sentinel cells of the immune system triggering inflammatory responses locally in the pre-cancerous niche^9^. In most cases, cytotoxic CD8^+^ T lymphocytes (CTL), effector cells of adaptive immunity, are the spearhead of anti-tumourigenic inflammatory responses. CTLs detect and subsequently eliminate tumours through the detection of tumour neoantigens that are presented to major histocompatibility complex (MHC) class I molecules on the surface of cancer cells. Individuals with tumours that are highly infiltrated by CTLs have been defined as ‘hot’ or ‘inflamed’ exhibiting a better overall survival and efficacy to T cell-based immunotherapies as opposing to ‘cold’ or ‘desert’ tumours that exhibit low CTL infiltration. The action of effector CD8^+^ T cells can be dampened by the presence of certain myeloid subsets (e.g. neutrophils and macrophages) and regulatory T cells (Tregs) by direct contact or release of pro-inflammatory mediators^18–20^.

**Figure 2:**
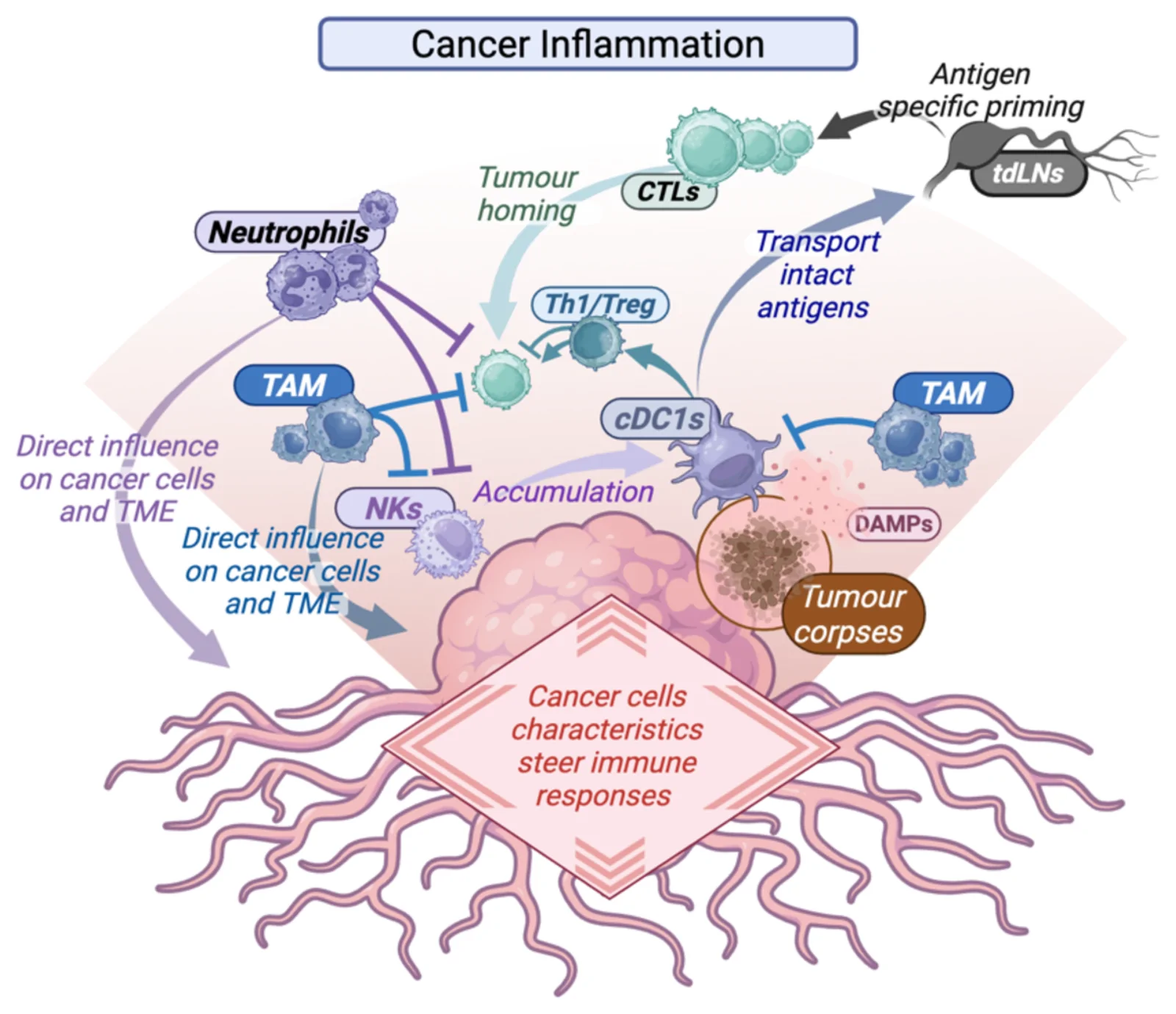
Schematic representation of the adaptive and innate immune responses in the tumour microenvironment.

Key to the instruction of T cell immunity is the acquisition of tumour neoantigens from cancer corpses by dendritic cells, sentinel cells of innate immunity. Cancer has been referred to as “wounds that do not heal” due to the increased prevalence of tumour cell death induced by tumour suppressor mechanisms or anti-cancer therapies. Further to tumour antigens, tumour corpses are a fruitful source of damage-associated molecular patterns (DAMPs) (DNA, F-actin, HMGB-1 etc.) that can bind to receptors found in phagocytes including dendritic cells, macrophages and neutrophils. The receptor-mediated signalling following DAMP recognition impact the activation status of phagocytes enabling them to uptake dead cell-associated tumour material and initiate an inflammatory response^8^ (Figure 2). Although they are limited in number within the TME, type 1 conventional dendritic cells (cDC1s) serve as the primary phagocytic cells responsible for transporting intact antigens to the tumor-draining lymph nodes (tdLNs) to facilitate antigen-specific CTL priming^21^. The activated CTLs will home the tumour by migrating towards chemoattractants CXCL9 and CXL10 produced, among other cells, by cDC1s in the TME^22^. In human cancers cDC1 levels have been associated with better prognosis, increased T cell intratumoral infiltration and enhanced responses to immunotherapy^23–25^. The role of cDC1 in anti-tumour immunity is controlled by their interaction with other immune subsets within the inflammatory milieu. XCL1 and Flt3L secretion by Natural Killer (NK) cells is required for the initial accumulation of cDC1s in the TME^23,24^. Furthermore, antigen-specific interactions between cDC1s and CD4^+^ T cell subsets, T helper 1 (Th1) or Tregs, can boost or blunt the ability of cDC1s to prime anti-tumour CD8^+^ T cells^26–29^ (Figure 2). Although cDC1s were shown to have an indispensable role in anti-cancer immunity, in few instances other myeloid subsets such as cDC2s and macrophages have shown to display non-redundant role in driving anti-cancer CD8^+^ T cell responses^30,31^.

Tissue resident and monocyte-derived macrophages outnumber cDCs in the tumour microenvironment and phagocytose apoptotic tumour cells rapidly limiting cDC access to antigens associated with the corpses^32^. Tumor-associated macrophages (TAMs) are often correlated with pro-tumourigenic inflammation and poor prognosis across various cancer types including those affecting the skin, brain, liver, and pancreas^33,34^. However, in certain cancers, such as colorectal cancer, TAM infiltration has been linked to better prognoses^33^. Indeed, a co-dependency between CTL exhaustion and TAM was observed, suggesting the potential use of myeloid presence in the TME as biomarker for immune-therapy responses^35,36^ (Figure 2). This highlights the clinical relevance of understanding TAM biology^33^. Clonal heterogeneity within tumours highlights the plasticity of macrophage activation towards pro- or anti-tumour phenotypes, with phenotypes varying based on their location within the tumour^33^. Further to the geographical region, the accumulation of the cytokine IL-4 was recently found to skew monocyte-derived macrophages towards a pro-tumorigenic phenotype^37^, supporting the idea that different innate immune cells are produced in the context of cancer, as will be discussed later in this review. Pro-tumorigenic TAMs dampen NK cell activity and drive T cell exhaustion through direct contact or secretion of cytokines (e.g. IL-1β, IL-10) that reprogram the TME^38,39^. In addition to their immunosuppressive function, tumour-resident IL-1β^+^ macrophages are associated with signatures of inflammatory reprogramming, epithelial-mesenchymal transition (EMT) and angiogenesis, further contributing to tumour progression^39^ (Figure 2).

It is also important to note that other myeloid or lymphoid cells can play dual roles in cancer-related inflammation. Neutrophils, for instance, have been shown to suppress T cells, remodel cancer cells and adjacent stroma and facilitate metastasis^40^ (Figure 2). Neutrophils were shown to undergo profound alterations within the TME, which instigate their tumour promoting functions^41^. On the other hand, neutrophils can exhibit anti-tumorigenic effects through presentation of tumour antigens to CTLs, highlighting phenotypic plasticity^42^. However, myeloid cells within the TME show an altered phenotype, which converge toward immunosuppression^43^. The role of B cells, unconventional T cells and innate-like lymphocytes in the TME is not as clearly defined and more work is needed to underscore their contribution in the control and progression of tumours at the primary site. Therefore, understanding the intricate regulation of immune cell interactions that determines whether inflammation will support anti-tumour immunity or promote tumour progression through immune evasion represents a priority question to improve cancer treatment.

##### Cancer shapes the inflammatory response

Despite the contribution of damage and infection, cancer cells themselves play a dominant role in sculpting inflammatory responses during tumour development. Tumour architecture, clonal heterogeneity of cancer cells, distinct geographical regions of different oxygen and nutrient supply in the TME and tissue specific cell-cell interactions generate local signalling hubs that promote continuous immune cell adaptation within tumour niches, favouring activation states that can favour or halt tumour evolution^8,9^.

Genetic instability and accumulation of mutations in malignant cells is a strong determinant of their inflammatory nature. High mutational burden in cancer correlates with increase neoantigenicity and increased intratumoral T cell infiltration and therefore it is considered a strong determinant of anti-tumorigenic inflammation. The clonality of tumour neoantigens will account for the total T cell repertoire influencing the overall immune response^9^. Although, tumours with high genomic instability exhibit increased neoantigen burden, they are also prone to accumulate mutations that enable them to adapt and shape the inflammatory milieu. Inactivation of tumour suppressors or activation of oncogenes have been previously shown to contribute to the types of inflammatory module present in established tumours^9^. For example, Myc activation drives progression of Kras^G12D^-induced pancreatic epithelial neoplasms (PanIN) to ductal adenocarcinomas (PDAC) accompanied by global transformation of the local stromal and immune landscape^44^. Tumour-derived factors as a result of aberrant oncogenic signalling, cancer cell state or altered metabolism might account for biases in inflammatory responses by impacting on immune cell plasticity and function, which can inhibit the anti-tumour activity of adaptive or innate immune cells^9^. Indeed, mutations or loss of p53 within cancer cells results in the production of cytokines that skews towards a pro-tumourigenic inflammatory microenvironment facilitating cancer cell invasion^45–47^. On the contrary, active β-catenin signalling reduces CCL4 production, hindering cDC1 infiltration and promoting cancer immune escape^48^.

Nutrients that are secreted or consumed by cancer cells as part of their metabolic needs can impact a variety of immune cell subsets including T cells, neutrophils and macrophages, reprogramming them towards a pro-tumourigenic state^9^. In addition, other tumour-derived factors contribute to immune evasion through suppression of conventional cDC accumulation and activation. The tumour-derived prostanoid prostaglandin E_2_ (PGE_2_) favours tumour progression by dampening anti-tumour immune responses pleiotropically^49,50^. PGE2-associated proinflammatory gene signature is negatively correlated with patient overall survival and response to immune checkpoint inhibitors^49^. Additionally, tumour-derived secreted-gelsolin (sGSN), an actin-scavenging molecule, plays a critical role in promoting cancer immune evasion by inhibiting the ability of cDC1 to prime CTL following recognition of F-actin exposed by cancer cells. Consistently, higher intratumoural levels of sGSN are associated with poorer overall survival outcomes in multiple types of human cancer^51^.

#### 1.1.2 Cancer Associated Fibroblast (CAFs)

**Figure 3:**
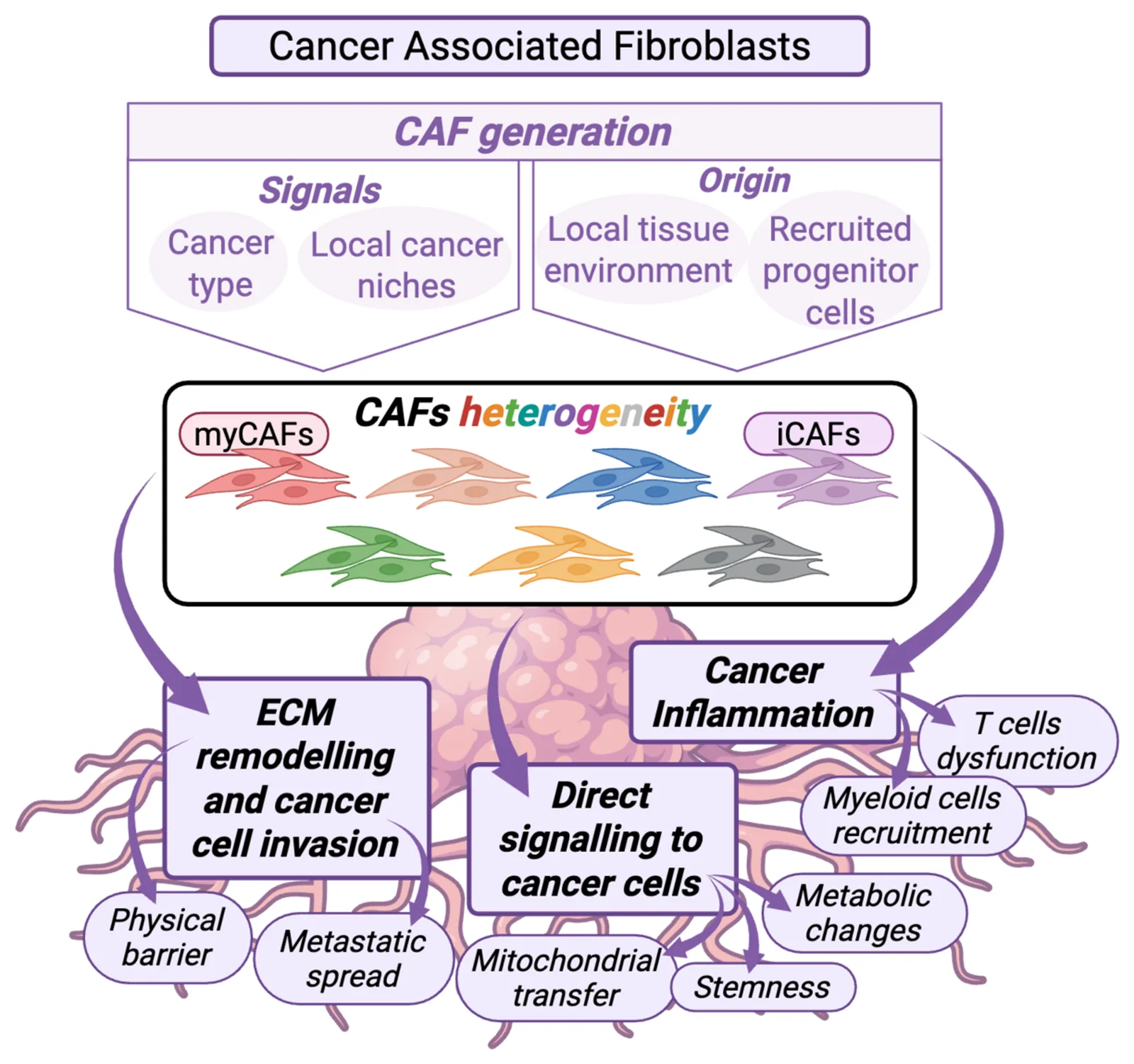
**Top panel:** A large diversity of cancer associated fibroblasts (CAFs) are generated within a growing cancer cells mass. CAFs heterogenicity depends on two factors the diverse signals activating them and their cell of origin. Diverse signals derive from an inter-tumour diversity between cancer types as well as from the intra-tumour diversity of various tumour microenvironment (TME) niches where different immune cells reside. CAFs can originate from different mesenchymal cells within the specific tissue of cancer origin or can be recruited on site from circulation. **Bottom panel:** Collectively the diverse CAFs drive various activity within the TME, influencing both cancer cells as well as other cellular and extracellular components.

CAFs have been recognised for many years as part of the TME in carcinomas, where in some cases, such as pancreatic cancer, they represent the dominant cellular component. Despite intense investigation, the origin of CAFs remains an open question, whose answer is complicated by the absence of distinctive lineage markers^52^. As for other cell types, the advent of single cell RNA-sequencing (scRNA-seq) has significantly enhanced our understanding of CAF heterogeneity^53^ (Figure 3). This heterogeneity arises, at least in part, on the diverse sources of CAFs and also depends on the presence of tissue-specific cells. Local tissue-resident fibroblasts are a main source of CAFs, with different subsets of normal tissue fibroblasts giving rise to functionally different CAF subtypes^54^. Additionally, the type and location of cancer influence the specific CAF profiles derived from tissue-resident fibroblasts. For example, CAF subtypes identified in cutaneous squamous cell carcinoma (cSCC) and originated from dermal fibroblasts show a distinctive profile from CAFs in basal cell carcinoma (BCC)^55^. Notably, CAF heterogeneity goes well beyond the heterogeneity of tissue-resident fibroblasts, since CAFs can also derive from a variety of other cell types, including bone marrow-derived mesenchymal stem cells, stellate cells, adipocytes, pericytes^5,52,53^. The extent to which each of these cell types contributes to CAF populations varies across tissues and may be influenced by the type of inflammatory cells enriched within different tumour regions^56^. The spatial organization of CAF subtypes within the tumour is increasingly recognized as a key factor in tumour progression, and changes in the CAF cluster distribution following disruption of mechanical forces impact on tumour growth^57^.

The initial identification of a dichotomy between more contractile myCAFs and more inflammatory iCAFs suggested the possibility that CAFs could exist across different states in cancer (similar to TAMs), and interconverting from one to another depends on specific signalling^58^. It is now clear that CAF heterogeneity extends well beyond this dichotomy, with the identification of several types of CAFs. To which extent these different subtypes are representative of different CAF states or depend on CAFs being originated from different cell types remain unclear. Notably, different stages of tumours can reflect different dynamics between CAF subpopulations, with iCAFs possibly enriched in growing late-stage tumours^59^.

CAF complexity is pushing the research community to provide a more accurate “catalogue” of CAFs, and define the different subpopulations based on their functional role in cancer^52,58,60^.

##### CAF functions

CAFs are well known for their ability to remodel the *Extra-Cellular Matrix* (ECM) and participate in cancer cell invasion and tumour spreading to metastatic sites^61^. Indeed, CAFs produce high amounts of ECM in tumours and this can enwrap cancer cells. This ECM capsule can then mediate cancer cell mechanotransduction and induce changes in tumour morphology^62^. Interestingly, CAFs also show heterogeneity, and somehow opposite effects, in their ECM remodelling abilities, with some patient-derived cells inducing a stiffening of the matrix while others promoting its softening^63^. Findings on the interactions between CAFs and ECM have been recently reviewed^64^. CAFs also promote tumour progression via *direct cross-talk with cancer cells*^65^. While this feature of CAFs has been known for many years, understanding CAF heterogeneity is helping to further dissect the molecular mechanisms by which they exploit this function. A subpopulation of TSPAN8^+^ CAFs, for example, promotes cancer cell stemness in breast cancer via the downregulation of SIRT6, mediating chemoresistance^66^. Moreover, new and surprising means of how communications between CAFs and cancer cells occur are still being discovered, such as the transfer of mitochondria from CAFs to cancer cells via the formation of tunneling nanotubes^67^. CAFs also promote cancer cell proliferation by inducing changes in cancer cell metabolism, for example by mediating the activation of glycogenolysis in cancer cells^68^ or by providing a direct source of nutrients^69–72^. Recent investigation has also identified sub-populations of CAFs critical for cancer cell survival, via paracrine IL6/IL8 secretion^73^.

In more recent years, the importance of CAFs in the *regulation of the inflammatory TME* has also emerged. CAFs mediate the recruitment of macrophages in the TME via their crosstalk with cancer cells and the consequent upregulation of HB-EGF/EGFR^74^. Given that CAFs are among the most abundant sources of secretory molecules in the TME, it does not come as a surprise that they can directly crosstalk with different components of the immune TME. CAFs support the formation of an anti-tumour immune environment^75^ by both promoting the recruitment of immunosuppressive myeloid cells^76,77^ as well as stimulating their tumour promoting functions^78–81^. Importantly, CAFs can also potentiate a pro-tumour immune response mediated by myeloid cells after chemotherapy or immunotherapy^81,82^. CAFs also impair adaptive immunity against the cancer cells by mediating T cell dysfunction and exclusion from the tumour core. While ECM released by CAFs could be a physical barrier to restrain T cell access to the cancer cell neighbourhood^83^, CAFs also prevent T cell trafficking in the TME or their activation via soluble factors^84–86^. CAFs have been mainly regarded as mediators of immunosuppression in the TME, and this has been thoroughly reviewed^87,88^. Notably, a greater understanding of CAF complexity is now favouring a better appreciation of immunostimulatory role of CAF subpopulations, particularly regarding their ability to support T cell activation^89^. This may be of particular relevance in lymph nodes, where fibroblastic reticular cells (FRCs) regulate B cell and T cell immunity. But it could also be critical at the tumour sites, where T cells, after the first activation in the lymph nodes, may need a “second touch” to become fully activated^90^. This could be mediated by antigen-presenting CAFs, a subpopulation of CAFs expressing MHC class II-related genes^91,92^.

#### 1.1.3 Vascular Response

##### Angiogenesis and lymphangiogenesis

Like normal tissues, all solid tumours also need vasculature for oxygen and nutrient supply. In physiological conditions, development of the vascular system involves both vasculogenesis and angiogenesis. The former refers to *de novo* formation of blood vessels, while the latter is the sprouting of new blood vessels from pre-existing vasculature, which occurs during development and wound healing. During tumour formation, the initial stage is avascular; however, when tumours grow beyond 1-2 mm in size, diffusion of oxygen and nutrients from surrounding tissue is no longer sufficient to sustain the cancer cells demands. The critical transition known as angiogenic switch^93^ will take place, leading to active recruitment of endothelial cells into the TME followed by new blood vessel formation. Although physiological and tumour angiogenesis share many common processes, the latter is characterized by a highly disorganized vascular network with increased endothelial cell proliferation, decreased pericyte coverage, abnormal basement membranes and increased permeability ^94^.

Angiogenesis is regulated by both pro- and anti-angiogenic factors. Notably, the former could be secreted by almost all the components of the TME and contribute to the angiogenic switch: tumour cells, immune cells^95^, CAFs^96^, pericytes and innervating nerves/neurons^97^. Classical pro-angiogenic factors include vascular endothelial growth factor-A (VEGF-A), a prototypic regulator of physiological and pathological angiogenesis, which signals through vascular endothelial growth factor receptor 2 (VEGFR2) and NRP1. Molecular mechanisms underlying tumour angiogenesis and angiogenic switch have been extensively documented in several classical reviews^98–101^. In parallel to angiogenesis, lymphangiogenesis can also occur during cancer development^102^. Among many pro-lymphangiogenic factors, vascular endothelial growth factor C (VEGF-C) and VEGF-D signalling through VEGFR2 and VEGFR3^102^ are the most characterized in pathological lymphangiogenesis^103^. One of the major functions of lymphatic vessels is to drain lymph into lymph nodes; when this route is hijacked by cancer cells, increased lymphangiogenesis is often associated with increased lymph node metastasis and worsened overall survival^103^.

##### Antiangiogenic therapy

**Figure 4:**
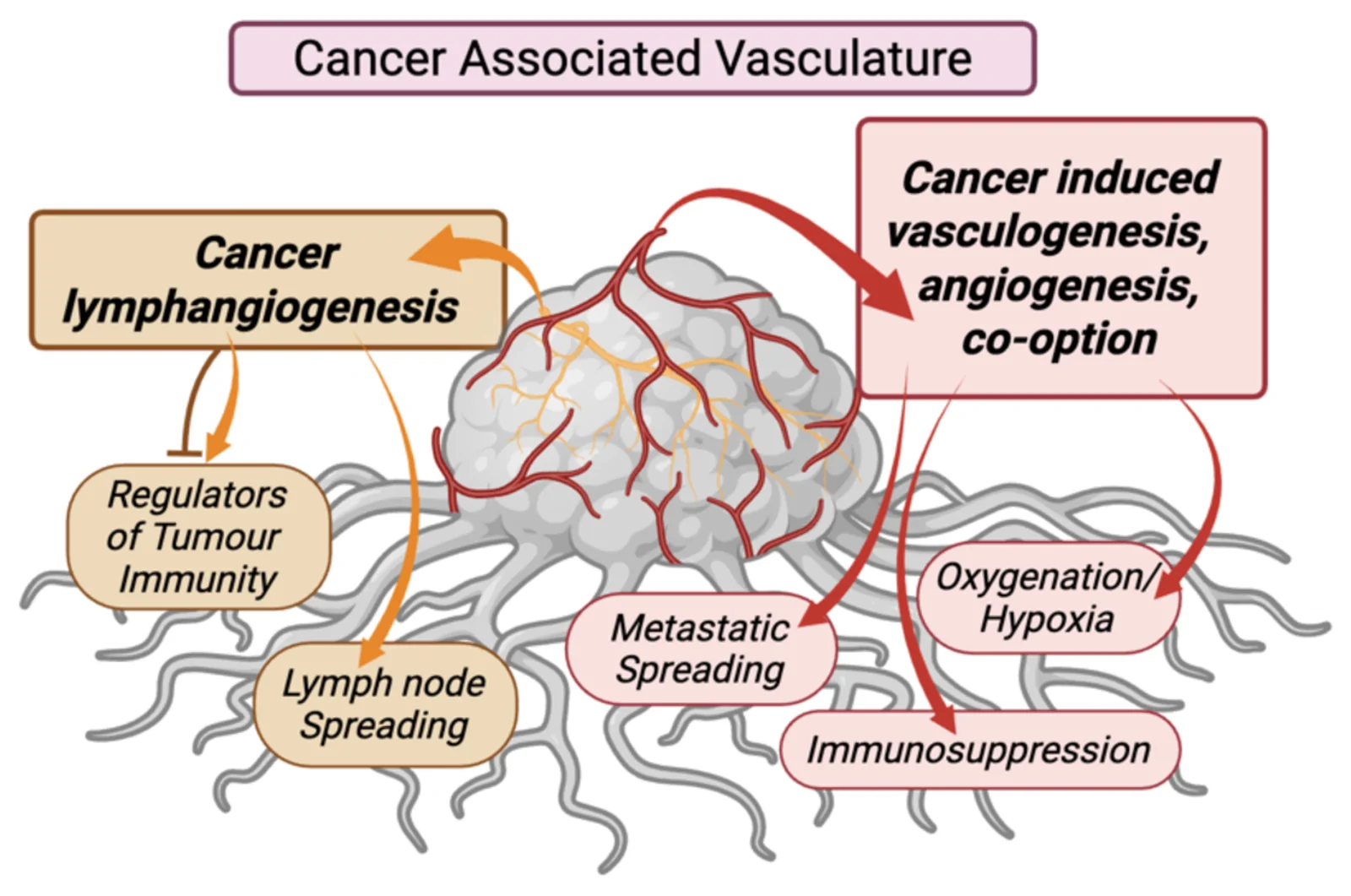
Cancer associated vascular response indicates the activities of cancer associated lymphatic vessels (on the left) and the ones of the cancer associated blood vessels (on the right).

The biology of angiogenesis has been extensively investigated since its discovery in the 70s by the late Judah Folkman^104^. His vision that angiogenesis is critical for tumour growth, therefore mitigating this process may be harnessed to treat cancer patients^104^, has led to the development of monoclonal antibodies^105^ and tyrosine kinase inhibitors (TKIs) targeting the VEGF pathway^106,107^. Notably, while the initial goal of anti-angiogenic therapy was to abolish blood vessel formation hence achieving tumour starvation and regression, the resultant, profound hypoxia was later found to activate hypoxia-induced factor (HIF) regulated genes, leading to evasive resistance, heightened aggressiveness and increased cancer dissemination^108^. Consequently, anti-angiogenic monotherapies often fail to provide long-lasting clinical benefit. On the other hand, combination of anti-angiogenic therapy with chemotherapy has led to improved clinical outcome in several cancer types^107^. Therefore, it was later proposed that, instead of completely blocking angiogenesis, anti-angiogenic therapies lead to “vascular normalization”^107,109^, which restores a more structurally and functionally normal vasculature and allows better delivery of cytotoxic drugs^110,111^. Notably, angiogenesis is highly associated with immunosuppression^112^: VEGF has direct immunosuppressive effects^113^, and immune cell trafficking is often impaired by abnormal tumour vasculatures^114^, via mechanisms including endothelial cell anergy^115^ (Figure 4). With the advancement of immunotherapies in recent years, combination of immunotherapy with anti-angiogenic therapy has also become an attractive approach to improve the efficacy of immunotherapy^107,115–117^. Similarly, lymphatic endothelial cells have also been recognized as direct regulators of tumour immunity^103^ (Figure 4). While tumour-associated lymphatic vasculature is reported to have immunosuppressive effects and limit anti-cancer immunity, paradoxically, lymphangiogenesis has also been linked to superior response to immunotherapies due to augmented transport of immune cells and antigens via lymphatic vessels^118^ (Figure 4). How the tumour-associated lymphatic vasculature could be targeted to synergise with immunotherapies remains to be further exploited.

##### Other mechanisms

In addition to classical angiogenesis, tumours can establish a vascular network through multiple non-angiogenic mechanisms^107^, including incorporation or co-opting existing vessels, especially in organs rich in blood supply^119^, and vasculogenic mimicry^120^, in which cancer cells form vessel-like structures to conduct blood. Cancer cells exhibiting vasculogenic mimicry tend to be highly aggressive and more metastatic. It is worth noting that these mechanisms are not mutually exclusive: the tumour microcirculation can be sustained through various mechanisms concommittently^120^, and this heterogeneity contributes to the therapeutic challenges to target angiogenesis in highly aggressive tumours (Figure 4).

#### 1.1.4 Tumour Innervation

The presence of nerves within tumours has been detected more than a century ago^121^. Since then, studies have identified different types of nerves innervating various cancers^122^. Co-culture of cancer cells and neurons demonstrated functional interaction between these two entities^123–126^, and manipulation of neural activity *in vivo* has also been shown to impact cancer progression.

However, in depth understanding of the role of innervation in cancer development has been hindered by technical challenges, as well as conflicting conclusions from different studies in the field. Several outstanding reviews have provided insights on the current knowledge and latest progress in understanding the interaction between cancer and the nervous system^127–132^. Since most progress in recent years in the cancer neuroscience field has been made in glioma^133–135^, brain metastasis^136^ and other central nervous system (CNS) cancers^137^, below we will focus on introducing basic concepts and techniques in neurobiology, and underscore their relevance in the research of solid tumours outside the CNS. We will also highlight the gap between current research and remaining crucial questions in the field.

##### Neurobiology and anatomy of the peripheral nervous system

The peripheral nervous system (PNS) consists of both the sensory (afferent) and motor (efferent) components. Afferent nerve fibers are axons of sensory neurons that relay information from sensory receptors to the spinal cord and brain. The motor system is subdivided into somatic and visceral motor systems; the former is responsible for voluntary movements of the muscle-skeletal system, while the latter is known as the autonomic nervous system (ANS), which can be further subdivided into the sympathetic, parasympathetic and enteric divisions^138^. All preganglionic autonomic neurons (both sympathetic and parasympathetic) are cholinergic. On the other hand, in the sympathetic system, postganglionic neurons use noradrenaline as the major neurotransmitter that acts on adrenergic receptors, whereas in the parasympathetic system, postganglionic neurons use acetylcholine as the major neurotransmitter to signal through cholinergic receptors^138^. The sympathetic, parasympathetic, and sensory components are most relevant for their interaction with most non-CNS cancers^139–143^, therefore will be our major focus of discussion here. Theoretically, the enteric nervous system (ENS) might also play an important role in gastroenteric cancers, although so far there has been little experimental evidence presented, hence will not be discussed herein. Detailed discussions regarding the role of ENS in other gastrointestinal diseases are available in several excellent reviews^144,145^.

Regardless of subtype, neurons are specialised for sending and receiving signals, and thus have distinct morphology compared to other cell types within our body. Typically, each neuron has three major structures: cell body, which contains the nucleus; dendrites, which receive signals, and axons, which conduct signals from the cell body. In the PNS, the cell bodies of neurons are located within structures called ganglia, with their axons extending into the tissues they innervate. Axons are bundled together with other axons and supportive cells to form peripheral nerves, which may extend to over a metre in length, such as those of the sciatic nerve traveling from the lower spinal cord to the foot.

Depending on the type of innervation, ganglia in the PNS are located either far away from or within innervated tissues: sympathetic ganglia are located close to the spinal cord; parasympathetic ganglia are located close to the effector tissues; finally, the cell bodies of somatosensory neurons are located within structures called dorsal root ganglia (DRG). Therefore, while nerve fibres can be detected within the TME, in most cancer types the cell bodies of neurons are not present in the tumour, making it challenging to investigate the innervation landscape either by bulk or single cell RNA-sequencing of tumours. In fact, tumour innervation has been mostly characterized and quantified by immunostaining so far^124,146,147^. In addition, neural tracing techniques^148^ have also been employed to characterize tumour innervation at the circuit level^149–153^.

##### Remodelling of the innervation landscape during cancer progression

It is likely that the innervation pattern is dependent on both the cancer type and the tumour stage. Notably, the diameters of the nerve fibres may vary from 0.2 – 1.5 μm in unmyelinated C fibres to up to 20 μm in myelinated motor neuron fibres. Considering that the typical thickness of parafilm slides is less than 5 μm, 100 – 200 μm thick cryosections and/or 3D tissue clearing techniques would serve to better resolve innervation patterns within the TME^147^.

**Figure 5:**
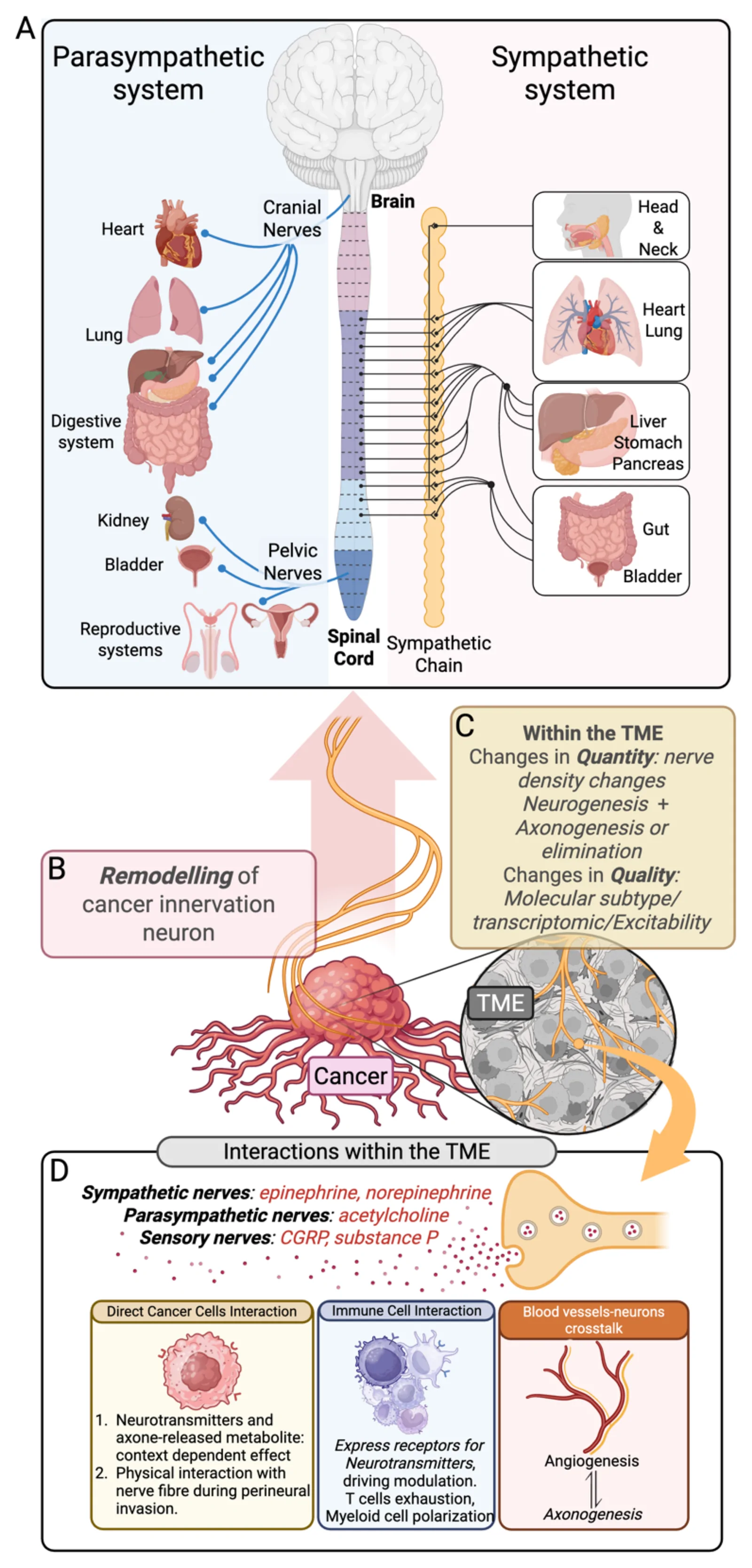
The complexity of the cancer-neural connection. A. Schematic representation of the two parts of the autonomic nervous system: the sympathetic and the parasympathetic arm, that together regulate bodily functions. B. Cancer innervation remodels its connection to the network of the autonomic nervous system. C. How innervation develops within the local Tumour Microenvironments (TME), via qualitative and quantitative changes to nerve fibre. D. Schematic representation of how various neurotransmitters can influence the behaviour of cancer and non-cancer cells within the TME harbouring neurotransmitters-receptors.

Alteration of the amount of nerve fibres within the TME has been frequently observed in various cancer types^147,154,155^. In addition to density changes, the molecular subtypes of innervation may also vary in distinct types of cancers, and are influenced by distinct driver mutations: Single cell profiling of traced neurons innervating normal pancreas and pancreatic ductal adenocarcinoma (PDAC) reveals significant remodelling of cancer-innervating neurons^153^; moreover, in head and neck cancer, loss of p53 has been shown to induce tyrosine hydroxylase (TH) expression in innervating neurons^125^, providing evidence that cancer genetics contribute to shaping the cancer-neuron crosstalk (Figure 5A and 5B).

It is also worth noting that since normal tissues are also innervated, detection of nerve fibres within the TME does not necessarily mean cancers have increased innervation. Quantitative comparison of innervation patterns between normal and tumour-bearing tissues at different stages would be informative to understand the role of nerves during different stages of disease progression^147,153,156,157^.

##### Neurogenesis and axonogenesis

Strictly defined, neurogenesis refers to the generation of new neurons from neural stem cells in the nervous system. In contrast, axonogenesis does not require the formation of new cells, but remodelling of the axons of pre-existing neurons. Both mechanisms have been identified in the TME: Prostatic ganglia are larger in size and harbour more neurons per ganglion in prostate cancer patients compared to those in cancer-free patients^158^. Moreover, doublecortin (DCX), a marker of neurogenesis in the central nervous system^159^, was found to be expressed in cells within prostate cancer to promote tumour progression^160^. Lineage tracing using *DCX-cre*^*ERT2*^ animals demonstrated that DCX signals co-localize with internexin and TH, markers frequently used to identify neural progenitors and adrenergic neurons, respectively^160^, suggesting neurogenesis within the TME during prostate cancer progression. On the other hand, axonogenesis, or axonal sprouting, has been suggested to play a role in PDAC^147,153^, because the number of innervating neurons remains relatively unchanged between normal and PDAC-bearing pancreas, while the axon coverage within the pancreas is increased in PDAC^153^. Regardless, despite their original definition in neurobiology, these two terms are sometimes used interchangeably in cancer literature, whenever increased nerve density is observed within the TME^154^. Notably, in addition to the alteration in innervation density during tumour progression, changes in the excitability of innervating neurones during cancer could also impact disease progression. Therefore, it would be important to assess both the quantify (nerve density) and quality (excitability) of innervation simultaneously at different stages of tumour development in future studies (Figure 5C).

##### The roles of nerves within the TME

Neuronal impulses are transmitted via specialised structures called synapses, which typically form either between two neurons, or between nerve and muscle fibres. There are two types of synapses: chemical and electrical. Chemical synapses constitute the majority of synapses in our body and operate via neurotransmitters released from the presynaptic cell to act on receptors located at the postsynaptic cell. On the other hand, electrical synapses are commonly observed in invertebrates and lower vertebrates, but much less so in the mammalian nervous system. Electrical synapses, whose synaptic cleft is only around 2nm, do not involve neurotransmitter release; instead, they function via gap junction coupling and are therefore bi-directional. In the CNS, it has been demonstrated that glioma cancer cells receive synaptic input from surrounding neurons and form functional circuits^161,162^; in addition, electron microscopy revealed a tripartite interaction among brain-metastatic breast cancer cells, neurons, and astrocytes, so that neuronally secreted glutamate activates NMDA receptors expressed on breast cancer cells to promote their growth within the brain metastatic niche^163^. Whether cancer-neuron synapses exist in the PNS remains to be explored. However, it is also worth noting that, in both physiological and pathological conditions, neurons/nerves can impact biological processes via both synaptic dependent and non-synaptic-dependent mechanisms. For example, postganglionic autonomic nerves use axon varicosities to engage effector cells, which do not necessarily possess specialised postsynaptic structures. Neurotransmitters secreted from nerve endings may also act on other non-neuronal cells expressing corresponding receptors in a paracrine manner^164^. Major neurotransmitters shown to play a role in cancer include: epinephrine and norepinephrine secreted by sympathetic nerves; acetylcholine secreted by parasympathetic nerve fibres; CGRP and substance P secreted by sensory nerves. Therefore, although a lot of attention has been drawn to the unexpected identification of cancer-neuron synapses in the brain, non-synaptic dependent interactions between tumour cells and innervating neurons are of no less importance and might have a broad(er) implication across different cancer types. Neurons and the neurotransmitters they secrete are known to play multiple roles and interact with various cellular components within the TME (Figure 5D):

##### Cancer cells

Classical neuronal receptors have been shown to be upregulated or ectopically expressed in various cancer types^136,156,165–168^. The ligand-receptor interactions between cancer cells and neurotransmitters secreted by innervating nerves have been more thoroughly explored in cancer types using genetically engineered mouse models (GEMMs), as shown in the following examples.

In mouse models of gastric cancer, CGRP secreted by nociceptive neurons^152^, as well as acetylcholine secreted by cholinergic neurons^169^, acts on RAMP1 or muscarinic acetylcholine receptor 3 (M3) expressed by cancer cells, respectively, to promote tumour progression. In PDAC GEMMs, adrenergic innervation promotes tumour progression via β2-adrenergic receptors expressed on cancer cells^140^, while cholinergic signalling, partly via vagus nerves signalling acting on the muscarinic acetylcholine receptor 1 (M1) expressed by cancer cells, suppresses tumour progression and prolongs survival^141^. Notably, completely opposite outcomes have also been demonstrated in other studies where sympathectomy accelerates tumour progression in another PDAC GEMM^147^ and hyperactivation of cholinergic signalling promotes orthotopic PDAC growth^170^. Indirect effects mediated by immune cells have been proposed in these studies. Moreover, high expression of M3 on cancer cells^171^ and increased VAChT+ parasympathetic nerve fibres within the stroma have both been associated with poor prognosis in PDAC patients^154^. Therefore, more systemic studies are necessary to reconcile the discrepancies among various studies from different research groups.

In addition to serving as the source of neurotransmitters, in a nutrient-deprived environment, axonally-secreted serine metabolically supports PDAC progression^172^, while CGRP secreted by sensory neurons induces cytoprotective autophagy in oral cancer cells^173^. Besides interacting with innervating neurons through secreted neurotransmitters, cancer cells can also physically engage nerve fibers within the TME via axon guidance molecules^174^ to promote motility^174^, or spread through perineural invasion (PNI), a phenomenon most commonly observed in head and neck cancers, PDAC, and prostate cancer^175^. Extensive crosstalk among cancer cells, nerves, Schwann cells, as well as other stromal cells, contributes to PNI^176–178^.

##### Immune cells

Neuro-immune interactions have been extensively investigated in various physiological and pathological conditions^179 164^, including skin infections, asthma and pneumonia^180^. Immune cells express a plethora of receptors for neurotransmitters^181^, which enable coordination with neuronal activity in response to changes in the environment, both external and internal. However, our understanding of the neuronal regulation of cancer immunity is only in its infancy. Some pioneering studies have just begun to examine various neuroimmune crosstalk within cancer settings: In melanoma, CGRP released by activated sensory neurons induces T cell exhaustion via RAMP1^124^, one of the major CGRP receptor components. In PDAC, sympathetic innervation induces T cell exhaustion via adrenergic receptors expressed on T cells^182^; in other studies, sympathectomy increased intratumoural CD163+ macrophages^147^, while hyperactivation of cholinergic signalling suppressed the intratumoural T-cell response^170^.

##### Endothelial cells

The vascular and nervous systems are closely linked both anatomically and developmentally^183^. Blood vessels are innervated by the perivascular ANS; various neurotransmitters released from nerve terminal varicosities help to set the vascular tone and regulate blood flow^184^. The crosstalk between nerves and endothelial cells is bi-directional; several axon guidance molecules and angiogenic factors are known to play a dual role in both systems, coordinating the patterning of nerves and blood vessels^183^. In prostate cancer, adrenergic innervation activates an angiogenic switch in endothelial cells via secretion of noradrenaline^97^; on the other hand, endothelial cells can also drive innervation in breast cancer via the axon-guidance molecule SLIT2^126^.

##### Neural manipulation impacts tumour progression

While increased innervation is often considered to promote tumour progression^172,185^, perturbations of neural activity have yielded inconsistent results in distinct cancer types, and sometimes, even in the same cancer type across studies from different groups, as mentioned in the previous section^141,154,170,171^. One of the plausible explanations is that various methods used in different studies may confound the interpretation of results; therefore, here we will summarize key methodology for neural manipulation in cancer models, focusing on their advantages and potential limitations:

###### Pharmacological and surgical method

Surgical denervation and pharmacological modulation of neural activity have been standard methods used to study the relationship between cancer and the nervous system in the past decades. Various α- and β-agonists and antagonists have been used to modulate sympathetic activity^186^, while cholinergic agonists and antagonists are used for parasympathetic modulation. At lower dosage, capsaicin, a vanilloid receptor TRPV1 agonist, activates sensory neurons; however, at high concentrations, capsaicin over-activates sensory neurons, leading to cell death, and has hence been used for sensory denervation in various models, including PDAC^139^, breast cancer^187^, melanoma^124^, etc. Sympathetic denervation can be achieved by 6-hydroxydopamine (6-OHDA)^142,147,157^ and adrenalectomy^140^; on the other hand, local injection of botulinum toxin type A^188–190^ as well as vagotomy^189^ are frequently considered for parasympathetic denervation. Importantly, it is worth noting that more than 80% of the vagus nerve is composed of sensory nerve fibres^191^; therefore, the effect of vagotomy should not be exclusively attributed to parasympathetic denervation. In addition, while these methods have provided important insights into the interactions between cancer and the nervous system, the lack of specificity and selectivity both in the location of perturbation and the neural subtypes perturbed may also complicate the interpretation of results.

###### Genetic methods

Modern genetic tools commonly used in neuroscience have been increasingly adapted by the cancer research community to investigate how neuronal activity impacts cancer progression. In optogenetic experiments, light-gated ion channels expressed in target neurons allow optical control of neuronal activity. Channelrhodopsin (ChR2)^192^, a light-gated cation channel, is frequently used to activate neurons with blue light. In contrast, halorhodopsin (eNpHR)^193^, a light-gated chloride pump, is commonly used to suppress neural activity. These tools allow for efficient manipulation of neural activity at millisecond resolution. On the other hand, chemogenetics are suitable for longer-term modulation of neural activity. Upon binding of synthetic ligands, chemogenetic DREADDs (*D*esigner *R*eceptors *E*xclusively *A*ctivated by *D*esigner *D*rugs) mediate prolonged activation (Gq-DREADD) or inhibition (Gi-DREADD)^194^ of the neurons expressing them. These methods have been widely applied to various neuroimmunology studies and will also allow cancer researchers to monitor and/or manipulate the activity of cancer-innervating neurons at a higher spatial, temporal and molecular resolution in the future. In addition to altering the activity of cancer-innervating neurons, these methods could also be employed to manipulate the electrical activity in excitable cancer cells^156^ and interrogate the subsequent impact on the cancer-nervous system interaction.

##### Context-dependent effects

While it might be tempting to assume that innervation is universally pro-tumoural, it is worth noting that manipulating neuronal activity may have distinct outcomes in different types of cancers^124,141,169,187,195^, suggesting that these effects might be tissue-specific^122^. Moreover, even for the same cancer type, manipulation at different stages of tumour progression may have different or even completely opposite results. For example, sympathetic nerves play a crucial role in the early development of prostate cancer in GEMMs, and parasympathetic innervation is important for late-stage tumour dissemination^142^. In a GEMM of PDAC, NGF blockage before tumour initiation promotes tumour progression, while it delays tumour growth when applied post tumour induction^196^. Therefore, the impact of cancer innervation may be highly complex and context dependent.

##### Cancer-neuroscience looking forward

Despite the identification of nerves within the TME over a century ago, technical limitations have been a major hurdle in advancing the knowledge of cancer-nervous system interaction. Fortunately, the technological breakthrough in the neuroscience field in recent years has provided the long-waited opportunity to revolutionize the field. In the past few decades, pilot studies in subcutaneous transplanted tumours have provided important insights in the interaction between cancer and the nervous system; in the future, various GEMMs of cancer, where cancers progress in their most native TME, would better inform the natural interaction between cancer and the nervous system at the relevant tissue niche, and would play an indispensable role in understanding the role of innervation in cancer progression.

#### 1.1.5 Tissue epithelial cells

**Figure 6:**
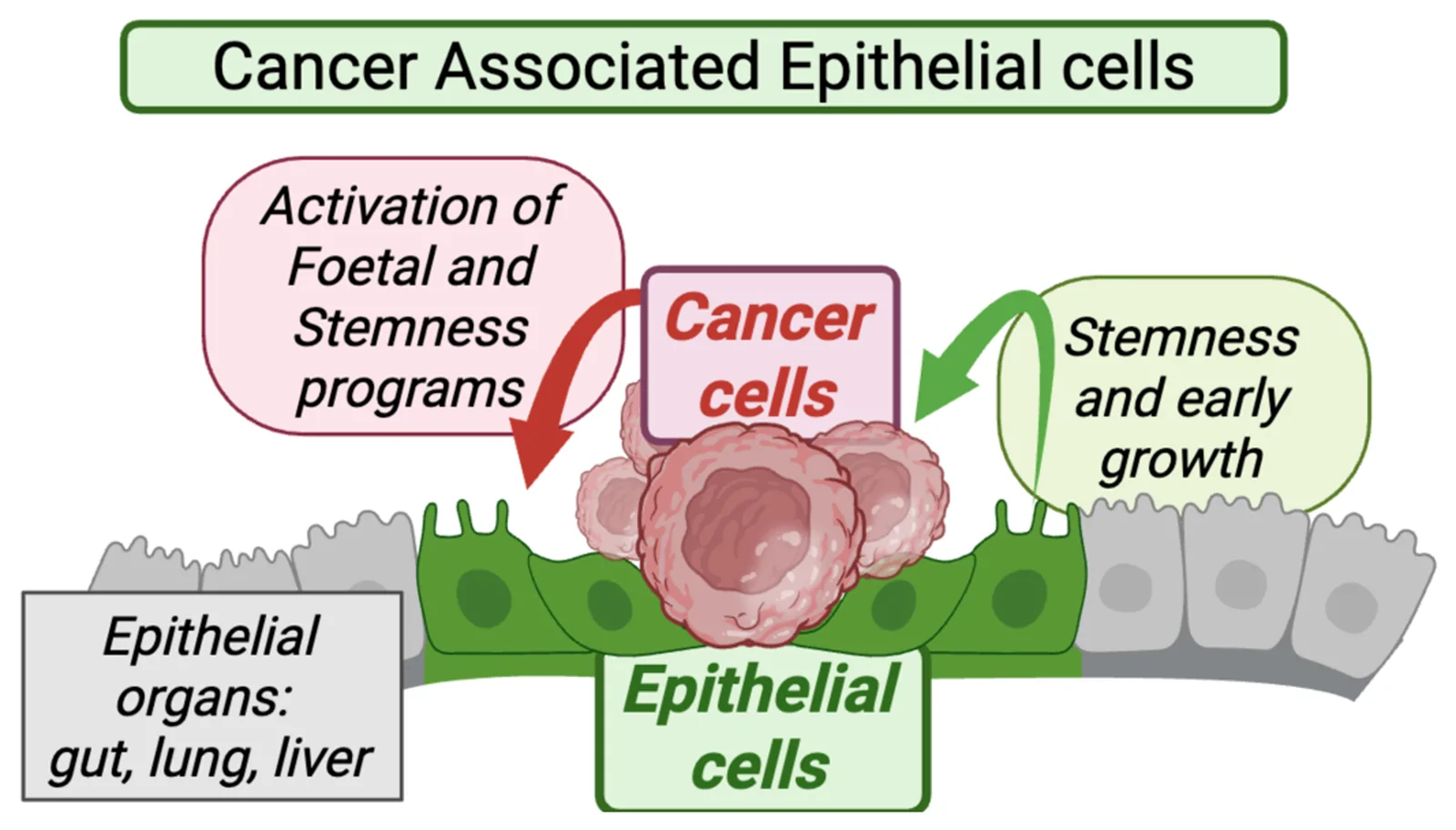
Schematic representation of the emerging cancer-epithelial interaction at early stages of cancer cell growth in epithelial organs.

Normal epithelial cells are another emerging component within the epithelial organ that are described to support the initial phases of cancer cell growth. Much of the supporting activities reported is linked to the activation of a regenerative program in the tissue parenchyma. In the context of colon cancer, a paracrine mechanism by which colon cancer cells activate a foetal program in normal intestinal cells was identified linked to YAP activation^197^. In oral squamous carcinoma, cancer cells were reported to directly induce a pre-cancerous behaviour in normal epithelial cells via delivery of cancer-derived EVs^198^. This phenomenon of cancer-epithelial interaction, especially at early stages of cancer growth in the tissue is better appreciated in the context of metastatic initiation. Here, by taking advantage of experimental strategies able to identify and study the cells in the metastatic organ establishing early contact with cancer cells, tissue epithelial cells were recognised to be an early component of the metastatic niche of lung and liver^199^. In the lung, alveolar cells were reported to directly engage with cancer cells and acquire a regenerative program leading to reacquisition of tissue stem/progenitor cell characteristics and the creation of a local environment that amplified breast cancer metastatic potential^200,201^. In the liver, a key role of hepatic cells was identified to support the seeding of disseminated tumour cells. Here plexin B2 on hepatocytes was shown to engage with class IV semaphorins on infiltrating colon cancer cells to activate KLF4 and support their growth^202^. These evidence highlights how normal tissue epithelial cell interactions support metastatic cells adaptation in the new organ, even before the establishment of their metastatic niche (Figure 6).

This suggest that for many epithelial cancers, an early engagement of epithelial cells surrounding pre-neoplastic mutated cells might be key in supporting transformation and cancer initiation.

### 1.2 Systemic perturbation caused from primary tumour

**Figure 7:**
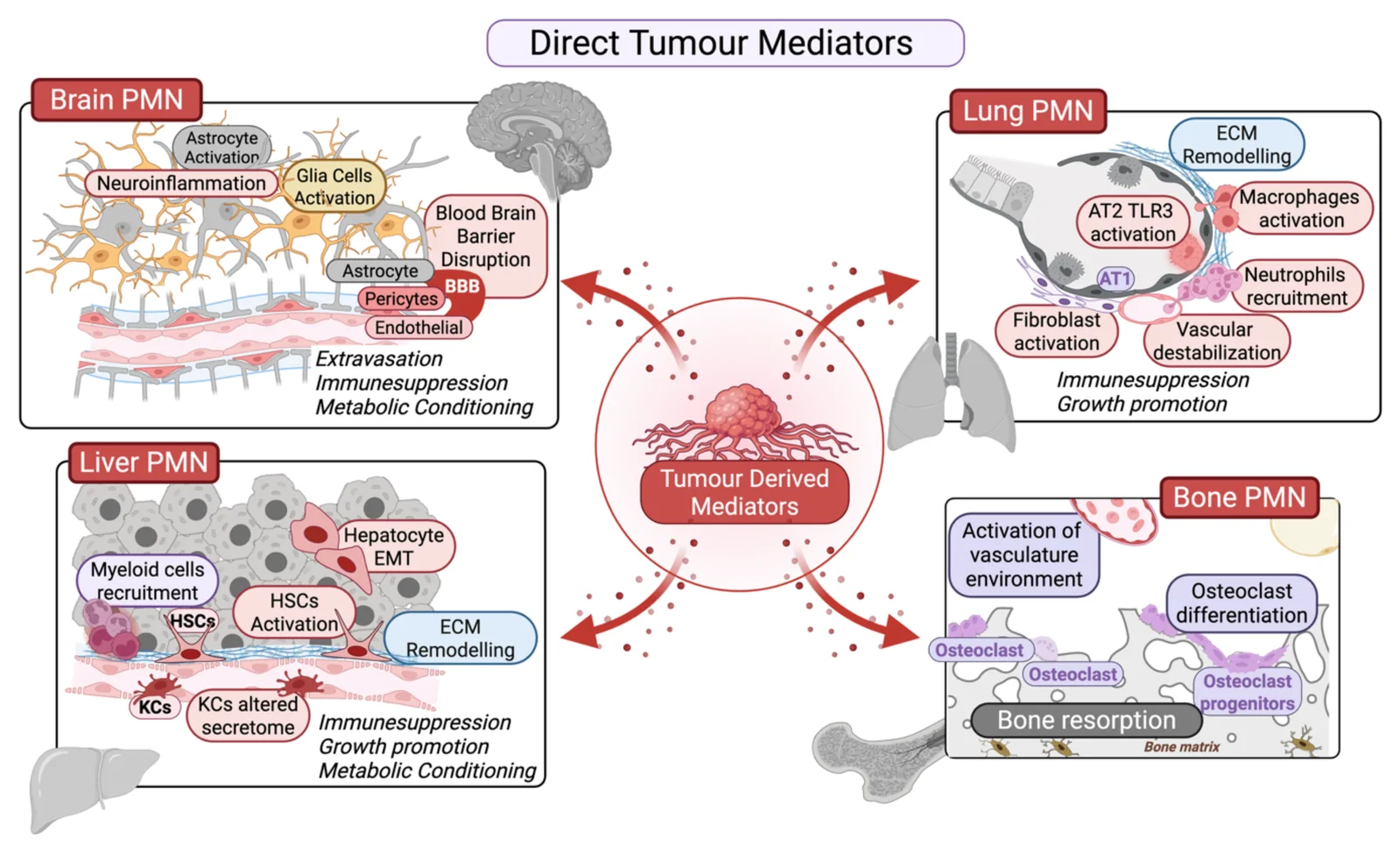
Schematic representation of how various mediators released by cancer drive systemic perturbations in distant metastatic organs - lung, liver, brain and bone - by directly targeting local tissue cells.

**Figure 8:**
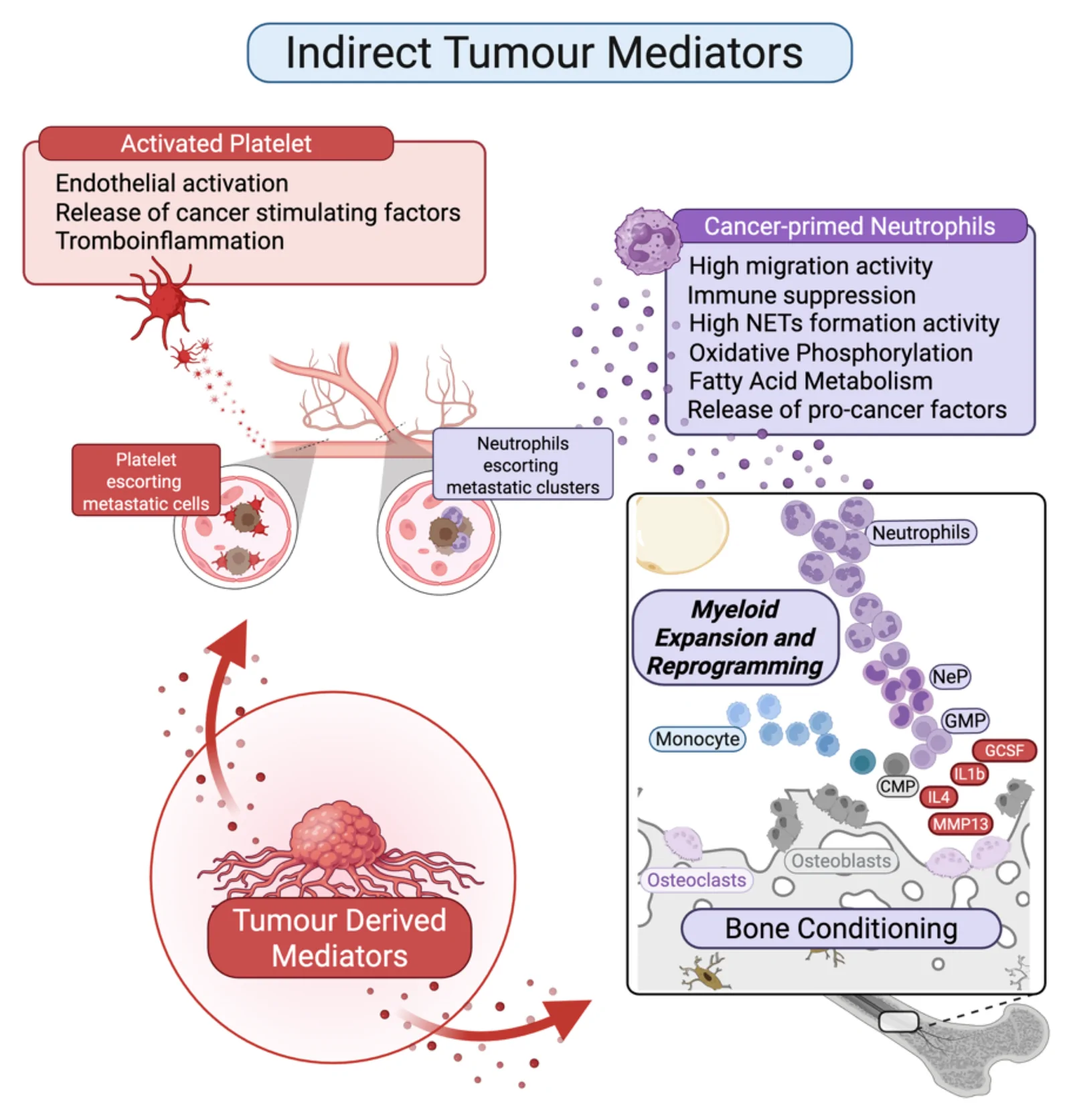
Schematic representation of how mediators released by cancer drive various systemic perturbations which influence neutrophils and platelets in circulation to alter distant organs.

As discussed above, the local response to emerging neoplastic cells is essential to build the heterogenous supporting structure of the tumour microenvironment, an integral part of growing and evolving cancers. However, these cancer-host interactions are not limited to the organ hosting cancer and impact many aspects of host physiology via the induction of systemic perturbations (Figure 1B). Those long distant interactions can occur by direct release of mediators from the tumour or indirect production of inflammatory mediators, or by organs in turn releasing factors that alter whole body physiology (Figure 7 and 8). For instance it was shown that many cancers induce alterations in the Urea cycle, with the consequent systemic pyrimidine imbalance resulting in a mutational bias and tumour antigens that increase immunotherapy responses^203^. Indeed, metabolic changes that at early stage of disease support cancer progression, such as the promotion of a pro-metastatic lipid enriched environment in the lung in the context of breast cancer^204^, become part of a self-amplifying loop that create more complex syndromes like cachexia^205^. It is therefore key to investigate not only how cancer cells themselves rewire their local metabolism, but also how systemic metabolic changes are induced from early stages and how they evolve over time, as these processes may represent key hubs for intervention (for a more detailed discussion see Peng-Winkler et al., 2026^206^).

The systemic conditioning of distant organs occurring at early stages of cancer favour metastatic progression in organs that will be targeted for metastatic dissemination, hence these perturbed distant sites are termed pre-metastatic niches (PMN). The relevance of those changes has been demonstrated clinically, in pancreatic cancer patients, where liver biopsies at the time of surgery were analysed for multiple characteristics including proliferation, presence of Neutrophils Extracellular Traps (NETs), and immune cells as well as overall signs of inflammation. Based on those changes, this multi-parametric profiling of liver biopsies was able to predict metastatic recurrence to the liver with high accuracy^207^.

This section will provide an overview of the different effectors mediating this systemic cancer to body conditioning.

#### 1.2.1 : Direct Tumour Mediators

Primary tumour cells secrete soluble factors and extracellular vesicles (EVs) that target healthy cells in distant organs to establish the PMN. These tissue-specific sites provide favourable conditions for disseminated tumour cells (DTCs), aiding their homing, adherence, survival and outgrowth. By conditioning and licensing tissues for future metastatic colonisation, tumour-secreted factors also play a key role in determining metastatic organotropism. Despite considerable heterogeneity and plasticity, there are commonalities in the pathological alterations associated with PMN formation including stromal activation and ECM remodelling; immunomodulation; rewiring of tissue-resident cells and disruption of vascular integrity, which have been recentlyreviewed^208^. In this section we summarise the various mechanisms of distant conditioning and tumour-secreted molecules that shape the PMN within the most common organs targeted for secondary metastatic growth: lungs, liver, brain and bone (Figure 7). Targeting these mediators may provide an opportunity to pre-emptively treat cancer patients prior to the onset of metastasis, likely to significantly influence patient survival.

##### Lung

###### Tumour conditioning of tissue-resident cells

A defining event in lung PMN generation is the activation of lung fibroblasts and induction of a fibrotic ECM, which in turn supports the adhesion and proliferation of incoming cancer cells. Tumour-secreted collagen crosslinking enzyme lysyl oxidase (LOX) is well established as a direct mediator of lung PMN formation^209^, and more recently, LOXL2 was shown to induce fibronectin, CXCL12 and MMP-9 production by lung fibroblasts, facilitating pro-tumorigenic ECM remodelling and recruitment of bone-marrow derived cells^210^. Upregulation of profibrotic genes and increased deposition of ECM proteins, in particular fibrinogen, are induced by tumour-secreted Activin A^211^, or via EV-mediated trafficking of TGF-β1^212^, IGF2BP1^213^ and Cav1^214^. Exosomes secreted by gain-of-function mutant p53-PDAC cells activates integrin trafficking in lung fibroblasts, provoking pro-invasive alterations in ECM organisation^215^. Notably, post-translational citrullination of fibrinogen deposits and their aggregation with serum amyloid A proteins in the lung PMN may create hotspots for the subsequent homing of metastatic cancer cells^216^.

Tumour-educated lung fibroblasts can also reshape the local immune landscape, building an immunologically tolerant PMN that favours metastatic outgrowth. Tumour-derived exosomes upregulate fibroblast secretion of CCL1, inducing Treg differentiation in the lung PMN^217^. Let-7 containing exosomes released by Lin28B^high^ breast cancer cells decrease immune surveillance via fibroblast-mediated recruitment of immune suppressive neutrophils^218^. Release of the pro-inflammatory IL-1β from breast cancer cells drives myeloid cell reprogramming via COX-2-expressing lung adventitial fibroblasts^219^ and can also trigger metabolic remodelling of the lung immune microenvironment^220^.

Lung alveolar epithelial cells exhibit immunomodulatory activity in response to primary tumour-derived signals. Tumour-derived exosomal RNAs activate TLR3 signalling in alveolar type II (AT2) cells, eliciting pro-tumoral neutrophil chemotaxis and lung PMN generation^221^. In osteosarcoma, tumour-secreted ANGPTL2 activates the integrin α5β1 receptor on AT2 cells and induces neutrophil accumulation^222^. Similarly, breast cancer derived EVs carrying miR200b trigger Ccl2 production by AT2 cells, supporting metastasis by driving recruitment of myeloid cells^223^. ScRNA-seq recently revealed a novel subpopulation of tumour-educated AT2 cells that express high levels of glutathione peroxidase 3 and support metastasis by dampening T cell responses^224^. Beyond immune regulation, tumour-mediated metabolic rewiring of AT2 cells increases palmitate availability in the PMN, fostering metastasis through enhanced NF-κB signalling^204^.

###### Immunosuppression

Tumour-derived factors can also directly influence inflammation and immune suppression in the lung PMN, enhancing the survival and engraftment of DTCs. Tumour-derived exosomes polarise tissue-resident interstitial macrophages towards a PD-L1+ immunosuppressive phenotype via NF-κB-mediated glycolytic metabolic reprogramming^225^. A similar phenotypic shift was observed following macrophage uptake of gastric cancer-derived exosomes enriched with miR-92a-3p, in this instance mediated by activated ERK signalling^226^. In osteosarcoma, EVs loaded with S100A11 stimulate lung interstitial macrophages via activated AKT/STAT3 signalling, which in turn enhance immunosuppression in the lung PMN via recruitment of neutrophils^227^. Osteosarcoma-derived exosomes have also been shown to induce pro-tumoral polarisation and increased production of TGF-β2 by alveolar macrophages^228^. Bone marrow-mobilised neutrophils are also well-established contributors to the lung PMN. Tumour secreted ANGPTL2 and protease Cathepsin C elicit pre-metastatic neutrophil recruitment and activation in the lung^222,229^, with the latter study demonstrating enhanced metastatic seeding due to the subsequent release of NETs^229^. Similarly, increased CXCL12 expression in PMN lungs of mammary tumour-bearing mice mediates trafficking of pro-tumorigenic CXCR4-expressing CD62L^dim^ neutrophils^230^. Beyond innate immune cells, gastric-cancer derived exosomes shape the immunosuppressive lung PMN by inducing T cell exhaustion and dysfunction^231^.

###### Vascular destabilisation

Corruption of the lung vascular barrier by tumour-secreted factors favours DTC extravasation and the influx of systemically mobilised immune cells. Tumour-medicated conditioning of premetastatic lung endothelium increases vascular permeability via upregulation of angiopoietin-2 and MMP3/10^232^ or downregulation of endothelial TRAIL by pro-angiogenic factors including VEGF^233^. Furthermore, neutrophil recruitment, cancer cell adhesion and outgrowth are enhanced through tumour-mediated hyperactivation of the Notch pathway in lung endothelium^234^. Sustained signalling through the Notch1 receptor triggers a senescence-like pro-inflammatory endothelial cell phenotype that facilitates tumour cell homing and trans-endothelial migration. Beyond the PMN, primary tumours can systemically perturb the body-wide vascular endothelium via upregulation of endothelial LRG1, perhaps as a mechanism to amplify pro-metastatic signals. The resulting boost in circulating Lrg1 promoted metastasis by expansion of lung perivascular cells^235^.

Destabilisation of the lung endothelial barrier can also be driven by exosomal trafficking of primary tumour-derived cargo, including tight junction-targeting miRNAs^236,237,238,239^; EV delivery of factors^240,241^. In an example of coordinated niche conditioning, the induction of a pro-metastatic endothelial niche is triggered indirectly by activated perivascular macrophages in response to tumour-secreted tenascin C^242^. CAFs can also serve as the intermediary between cancer cells and the pre-metastatic lung vasculature, with uptake of tumour-secreted lncSNHG5 by lung stromal cells invoking vascular destabilisation^243^.

##### Liver

In response to primary tumour secreted molecules, hepatic niche preparation occurs via the coordinated actions of tissue-resident cells including hepatic stellate cells (HSCs), Kupffer cells (KCs) and hepatocytes, which together create a fibrotic and pro-inflammatory milieu that supports metastatic colonisation.

###### Stromal activation

Activation of HSCs is a hallmark feature of the liver PMN, facilitating metastasis via ECM remodelling, chemokine secretion and immune modulation. In colorectal cancer liver metastasis, the deposition of fibrotic ECM by HSCs is triggered by exosome-mediated delivery of miR-181a-5p^244^ or the pro-oncogenic molecule ITGBL1^245^. Similarly, PDAC-derived exosomes containing the CD44v6/C1QBP complex or transfer RNA-derived fragment Trf-GluCTC-0005 can directly activate HSCs and induce liver fibrosis and infiltration of myeloid cells^246,247^. The enhanced deposition of ECM components, primarily fibronectin but also collagens, laminins and proteoglycans, increase the efficiency of metastatic seeding, with liver fibrosis known to increase tumorigenicity through increased integrin signalling, enhanced ECM stiffness, growth factor liberation and recruitment of immunosuppressive cells^248^.

HSC activation can also be triggered indirectly via interplay with other tumour-conditioned niche cells. In an elegant example of coordinated PMN preparation, PDAC-exosomes loaded with macrophage migration inhibitory factor (MIF) induce TGF-β secretion by KCs, the predominant resident macrophage population in the liver, which in turn triggers HSC activation and fibronectin deposition^249^. The fibrotic ECM augments liver metastasis through recruitment of bone marrow-derived macrophages. KCs can also induce TGFβ-mediated HSC activation following uptake of gastric cancer secreted LPS binding protein^250^. Hepatocytes orchestrate HSC activation in response to PDAC-associated stromal IL6, via activation of hepatocyte STAT3 signalling and release of serum amyloid proteins^251^. The resulting pro-fibrotic alterations and immune suppression in the liver condition the organ for incoming cancer cells.

###### Immune Modulation

Cancer-educated HSCs construct an immune suppressive niche via secretion of pro-inflammatory cytokines and leukocyte recruitment. Colorectal cancer-derived exosomes enriched in TGF-β1 trigger HSC activation and CXCL12 secretion, which in turn promotes myeloid cell recruitment and inhibition of NK cell cytotoxicity in the PMN^252^. Systemic elevation of Tissue Inhibitor of Metalloproteinases (TIMP)-1 is a key driver of liver PMN formation via enhanced neutrophil chemotaxis, with elevated serum TIMP1 corresponding to poor prognosis across multiple cancer types^253^. In response to PDAC-secreted TIMP1, activated HSCs recruit pro-metastatic neutrophils via secretion of SDF-1^254^. Importantly TIMP1-mediated niche conditioning is also instigated by pre-malignant PanIN lesions, a phenomenon perhaps reflected in the high efficacy of PDAC liver metastasis.

Cancer-mediated conditioning of KCs helps shape the immunosuppressive PMN. Tumour exosomes elicit pro-inflammatory alterations in the KC secretome, such as the release of S100A8/P molecules following uptake of exosomal ITGα_v_β_5_^255^ or miR-21-driven secretion of IL6 via activated TLR7 signalling^256^. Wang et al. demonstrated that colorectal cancer-secreted VEGFA stimulates tumour-associated macrophages to produce CXCL1. Increased circulating CXCL1 triggers the influx of CXCR2+ myeloid cells to the hepatic PMN, supporting metastatic seeding^257^.

The induction of immune tolerance in the liver PMN, characterised by pro-metastatic macrophage polarisation and suppression of hepatic NK cells, can be induced by breast cancer cell-secreted stress response protein GRP78^258^. Colorectal cancer-derived exosomes encapsulating miR-934 or miR-203a-3p induce M2 macrophage polarisation in the PMN through activation of PI3K/Akt signalling, fostering metastatic seeding via secreted CXCL12/CXCL13^259,260^. Phenotype switching in intrahepatic macrophages can also occur following uptake of cancer derived exosomal miR-519a-3p, augmenting metastasis via secretion of pro-angiogenic cytokines^261^. Finally, exosome cargo can dampen T cell responses in the liver PMN, with miR-135a-5p-mediated reduction of IL2 and TNFα secretion by KC cells resulting in supressed CD4^+^ T cell recruitment and activation^262^.

###### Metabolic Conditioning

Tumour secreted molecules can alter growth factor signalling and nutrient availability in the liver PMN, directly supporting cancer cell seeding and outgrowth. Colorectal cancer-derived exosomal HSPC111 alters lipid metabolism in HSCs by elevating intracellular accumulation of acetyl CoA. This in turn promotes HSC secretion of pro-tumorigenic CXCL5 via H3K27 acetylation, enhancing cancer EMT and augmenting liver metastasis^263^. Generation of a microenvironment rich in secreted hepatocyte growth factor (HGF) promotes proliferation, invasion and migration of incoming cancer cells. In colorectal cancer, this occurs via exosomal transfer of miR-221/222 from tumour cells to HSCs, similarly gastric cancer-derived exosomes loaded with EGFR elicit HGF secretion following uptake by both KCs and HSCs^264^. Lung adenocarcinoma EVs trafficking lncRNA-ALAJHM also promote hepatocyte secretion of HGF^265^. In an example of niche and tumour co-adaptation, PDAC-induced secretion of SLIT2 by hepatocytes supports the proliferation and survival of incoming cancer cells expressing receptor ROBO1 ^266^. Generation of a pre-metastatic environment rich in TGF-β1 also supports tumour seeding by enhancing the stemness properties of newly arrived cancer cells. In gastric cancer liver metastasis, exosome-mediated delivery of miR-151a-3p to KCs initiates a stemness-enhancing niche via TGF-β1/SMAD2/3 pathway activation, supporting self-renewal and EMT^267^. Tumour-educated hepatocytes also undergo TGFβ-1/SMAD-mediated EMT in response to colorectal cancer EVs^268^.

##### Brain

The unique anatomical and metabolic constraints of the brain microenvironment create a hostile soil for disseminating cancer cells, thus tumour-mediated conditioning of the neural microenvironment is a prerequisite for successful metastatic colonisation.

###### Modulation of the brain vascular niche

Disruption of the blood brain barrier (BBB) is a key event during neural PMN generation. Exome-mediated delivery of CEMIP from brain-trophic breast cancer cells to neural endothelial cells and microglia fosters metastasis through pro-inflammatory cytokine secretion, vascular remodelling and loss of BBB integrity^269^. Primary tumour CEMIP may serve as a prognostic biomarker of brain metastases and poor survival in patients. Vascular integrity is compromised by NSCLC-derived exosomes carrying lnc-MMP2-2 via downregulation of endothelial miR-1207-50 and disruption of tight junctions^270^. Dissolution of BBB function is similarly induced by breast cancer-derived EVs carrying miR-181c, miR-105 or exosomal lncRNA GS1-600G8.5, which modulate the localisation of endothelial cell actin filaments and destroy tight junctions, enhancing extravasation^236,271,272^. More recently, EV-trafficked miR-374-5p secreted from NSCLC promoted leptomeningeal metastasis via loss of tight junctions and BBB integrity^273^.

###### Induction of neuroinflammation

Neuroinflammation is characterised by enhanced pro-inflammatory cytokine/chemokine secretion, persistent activation of astrocytes and microglia, immune suppression and vascular destabilisation^274^. Although neuroinflammation in cancer has largely been studied in the context of established metastatic lesions, its role in the PMN is emerging. Lung cancer derived soluble factors IL-8 and MIF elicit astrocyte activation *in vitro* and secretion of pro-tumorigenic mediators IL-1β, TNFα and IL6^275^. More recently, EV-mediated transfer of LINC00482 from NSCLC to microglia was shown to elicit TGF- β1-mediated M2-like polarisation, fostering an immunosuppressive PMN and potentiating brain metastasis^276^. NSCLC-derived exosomes can also mediate microglia activation and pro-tumorigenic polarisation following uptake by ECs, stimulating release of Wnt pathway inhibitor Dkk-1 to nearby microglia^277^. Interestingly, EVs secreted by brain-trophic breast cancer cells aggregate with low-density lipoprotein, enhancing their uptake by immunosuppressive brain monocytes^278^.

###### Metabolic conditioning

Rewiring of energy metabolism in both the brain microenvironment and colonizing tumour cells is a key feature of the metastatic niche^279^, however the specific metabolic adaptations in the pre-metastatic phase remain largely unknown. Fong et al elegantly demonstrated enhanced metastasis through glucose reallocation in the brain PMN, since EV transfer of breast cancer-derived miR-122 to astrocytes reduces glucose consumption via downregulation of GLUT1, favouring metabolism of incoming cancer cells^280^.

##### Bone

###### Disruption of bone homeostasis

Tumour-secreted molecules can tip the balance of bone homeostasis in favour of bone resorption, leading to osteolytic pre-metastatic lesions that serve as a highly supportive niche to foster colonisation of newly arrived DTCs. A wealth of soluble factors that induce osteoclast differentiation and activation have been described, including LOX, PTHrP, OPN, IL-6 and IL-8^281^. More recently, enrichment of osteoclast progenitors and preparation of the osteoclastic PMN was demonstrated in response to breast cancer-secreted DKK1 and suppression of canonical Wnt signalling^282^. The activation of osteoclastic bone resorption subsequently triggers the release of factors such as insulin-like growth factor-1 and bone morphogenic proteins from the bone matrix, supporting the construction of a cancer-supportive niche.

In addition to soluble factors, primary tumour-secreted exosomes can modulate osteoclast activity, for example delivery of miR-21 to osteoclast progenitors which promotes osteoclast differentiation and activation^283,284^, or via exosomal-delivery of amphiregulin, which induces osteoclast differentiation through activation of the EGF pathway^285^. Exosomal miR-19a and Integrin-Binding Sialoprotein (IBSP) secreted by bone-trophic breast cancer cells co-operatively regulate PMN formation, with IBSP stimulating recruitment of osteoclast progenitors while miR-19a promotes osteoclastogenesis via NF-κB/Akt pathway activation^286^. Tumour-derived signals can also indirectly trigger osteolysis, with EV-mediated shuttling of miR-378a-3p from prostate cancer cells to bone marrow macrophages promoting osteolysis via upregulation and secretion of Angptl^287^.

The survival and colonisation of disseminated tumour cells in the bone microenvironment can also be supported by osteoblastic/osteogenic pre-metastatic niches, particularly in prostate cancer metastasis. Prostate-cancer derived exosomes carrying hsa-miR-940^288^ or miR-141-3p^289^ promote osteoblast differentiation and activity, augmenting osteoblastic lesion development *in vivo*. Breast cancer-secreted EVs carrying osteoblast cadherin (CDH11) and ITGA5 cooperatively educate osteoblasts to establish an osteogenic PMN that in turn supports the invasive spread of incoming cancer cells^290^.

###### Modulation of immune and vascular niches

Metastatic colonisation by bone-trophic breast cancer cells is associated with the induction of an immune suppressive milieu in the bone PMN, characterised by an increase in myeloid cells and reduced T cells, dendritic cells and NK cells^291^. A subset of breast tumour-educated CD4+ T cells expressing RANKL accumulate in the bone PMN where they activate osteoclast differentiation, possibly via dendritic cells as osteoclast precursors, resulting in metastasis-promoting osteolytic lesions^292^. Interestingly tumour priming of CD19+ B cells potentiates this process, with T and B cells from tumour-bearing mice cooperating to induce pre-metastatic osteolytic disease^293^. The bone vasculature is another important player in niche formation, with DTCs from murine mammary tumours preferentially seeding within a specialised endosteal vascular niche enriched in type H vessels. Subsequent pro-tumoral vascular remodelling facilitates lesion outgrowth^294^.

#### 1.2.2 Indirect mediators: Circulating cells

##### Neutrophils

Alongside the induction of a bone PMN mediated by the mechanisms described above, alterations in the bone niche have another important consequence on neutrophil production and mobilization (reviewed^40^), inducing systemic alterations more indirectly (Figure 8). Higher number of neutrophils were reported to infiltrate distant organs and represented one of the first drivers of the distal PMN^295,296^, and a large body of literature highlights the many activities of neutrophils in the context of the cancer program (reviewed^297,298^). However, in this review, which mainly focuses on the cancer-induced body perturbation, a relevant concept to emphasise is that inflammatory signals coming from the primary tumours’ environment such as IL1β^46^ and granulocyte-colony-stimulating factor (GCSF)^296,299^ increase and strongly alter granulopoiesis^300^. Inflammatory mediators are not only released by activated cells within the TME^5^, but also by cancer cells. This could depend on specific cancer cells’ characteristics, such as their genetic instability leading to cytoplasmatic DNA, which promotes senescence via cGAS-STING causing the release of pro-inflammatory signals^301^. Together with the perturbed bone marrow niche described above, all those inflammatory mediators influence hematopoietic progenitor cells differentiation, inducing alterations in myelopoiesis. Indeed, in the presence of cancer, the expansion of unipotent neutrophil progenitors was observed in the bone marrow of mice and humans^302^. This altered myeloid production depends on alterations in hematopoietic stem cells, but also in their mesenchymal niche^303^. A recent study on lung cancer showed that alterations in bone marrow cells causes local release of IL4 by basophils, which acts on granulocyte-myeloid progenitors (GMPs) and transcriptionally reprograms the progeny of these lineages entering the circulation toward a pro-tumorigenic phenotype^37^. Therefore, not only is neutrophil production increased in the context of cancer, but cells are born with different characteristics (cancer-primed neutrophils). Another example is that EVs from different solid cancers were shown to increase osteoprogenitors, inducing GMPs aggregation and increased production of immune-suppressive myeloid cells^304^. Most interesting, this phenomenon persists after tumour removal suggesting that those alterations in the innate immune cells might have a more lasting effect than initially anticipated^304^. Similar alterations in neutrophil characteristics in murine cancers are well documented in human patients^305,306^. A recent study showed how these perturbations in circulating neutrophils in newly diagnosed breast cancer patients is detected at very early stage of disease and that differ between cancer types^307^. Cancer-primed neutrophils are described to have many pro-tumour functions, the most characterised of which being *immune-suppression* so much so that many reports refer to neutrophils as myeloid-derived suppressor cells (MDSCs)^305^. MDSCs is a general term used to define cells of the myeloid lineage (monocyte and neutrophils) which display immune-suppressive functions in the context of cancer. A study using murine model of lung cancer at different stages showed an evolution in cancer-primed neutrophil’s characteristics: with high neutrophil migration and oxidative phosphorylation activity more typical at early-stage cancer and immunosuppression emerging at later stages^308^. Stress signals are also reported to be important for the pro-tumorigenic activity of neutrophils and involved in their cancer-primed characteristics^309,310^.

Despite the substantial reprogramming neutrophils undergo once they infiltrate the tumour, their immune-suppressive function plays a key role within the TME and influences response to immunotherapy^311,312^. Moreover, the presence of interferon stimulation within the TME profoundly influences immunotherapy responses^313,314^. Cancer-primed neutrophils also show a different metabolism including fatty acid metabolism^315^ and mitochondrial metabolism^316^ which supports immune-suppression. Neutrophils can also *directly support metastatic cell* survival in circulation as well as their tumour initiation potential^296,317,318^.

Another important neutrophil function impacting on cancer is *NETosis*. NETs are web-like extracellular structures of DNA, histones, and cytotoxic granule-derived proteins that instigate many changes in the tissue including *ECM changes*, immunosuppression and even direct cancer cell stimulation^319–322^. Similarly to their immunomodulatory function, NETs released by neutrophils were found to be induced upon chemotherapy and confer therapy resistance^323^.

Moreover, an elegant study demonstrates that, due to their ability to induce changes in the organ they infiltrate, neutrophil migration into the liver causes changes in the host metabolism^324^. Here, in a model of breast cancer, this immune infiltration causes the depletion of a master metabolic regulator, HNF4α, leading to metabolic changes that promote breast and pancreatic cancer growth and worst outcome.

Despite the large body of literature supporting the idea that cancer-primed neutrophils are pro-tumorigenic, and their cytotoxic activity remain suppressed, evidence of their cancer killing functions also exist^325,326^. Therefore, it is tempting the idea that this potential could be exploited as an immunotherapeutic. Proof of concept studies exists where neutrophil stimulation shows their tumour-eradication potential *in vitro* and *in vivo*^327,328^.

By the variety of the above-mentioned functions, cancer-primed neutrophils contribute to both the formation of a tumour supporting microenvironment and, by systemically infiltrating and conditioning distant organs, the formation of pre-metastatic niches (reviewed^208^). Moreover, they can interact with cancer cells in circulation to support their pro-tumorigenicity^329^. Another partner of circulating neutrophils are platelets, an interaction known to be significant in the context of inflammation^330^ as well as tromboinflammation, which represents a leading cause of death for cancer patients^322,331,332^.

##### Platelets

Platelets, which are also reported to interact and directly support circulating cancer cells^333^, can be increased in cancer patients and associated with higher mortality^334^. Platelets contribute to the formation of pre-metastatic niches favouring endothelial cell activation and the recruitment of pro-tumourigenic monocytes/macrophages^335^ (Figure 8). They can also directly interact with cancer cells and increase their metastatic ability. For instance, platelet-derived autotaxin, which controls levels of lysophosphatidic acid (LPA) in blood, was found to support breast cancer metastasis to the bone^336^. Moreover, cancer-activated platelets were shown to induce, via P2Y_2_ receptor on endothelial cells, the endothelial permeability, promoting cancer cell extravasation and lung metastasis^337^. P2Y_12_ receptor on platelets was shown to be important for platelet-cancer cell interactions and depletion of P2Y_12_ on platelets reduced growth of orthotopic ovarian tumours^338^. Also, TGF-β1 derived from platelets was described to support ovarian cancer growth^339^.

As we will discuss in the second part of this review, there are various external factors that by influencing the body physiology increase the chances of cancer progression. Platelets can mediate this phenomenon. Indeed, in the context of a high fat diet (HFD), a pre-activation of platelets and endothelial cells was observed, which enhanced lung metastasis via increased vascular leakiness, overexpression of fibronectin and interactions between platelets, tumour cells and endothelial cells^340^.

Besides the various mechanisms whereby platelets impact cancer cell growth and metastatic potential (reviewed^341^), another critical consequence of the increased level of platelets, leukocytes and tissue factor (TF) in cancer patients is represented by the *thromboinflammation* leading to increased risk of venous thromboembolism^342–344^ and heart failure^345^ in advanced stages of the disease. This is part of the systemic cancer-associated alterations that can cause acute events leading to mortality^332^. In lung cancer, which was found to associate with all coagulation abnormalities, coagulation indexes were suggested as potential measure for timely disease management^346^. Those can lead to an abnormal response of the organism to cancer treatments. Indeed, thrombocytopenia is a common side effect of chemotherapy in solid cancers caused by a drop in platelet counts and higher risk of bleedings^347^.

In summary, platelets engage with cancer at different levels and from early to the late stage of the disease. As mentioned above, their production is increased in cancer, but also their RNA content, which, as per their nature, is strongly influenced by extrinsic cues and thus is altered by cancer. A remarkable clinical study demonstrated that RNA-based blood tests of cancer-educated platelets at early and late disease stage, was able to detect 18 cancer types and their tissue of origin with high specificity^348^. This study highlights how profoundly platelets are perturbed in the cancer context and proposes the use of platelet RNA analysis as a powerful cancer screening approach.

### 1.3 The advanced cancer disease

**Figure 9:**
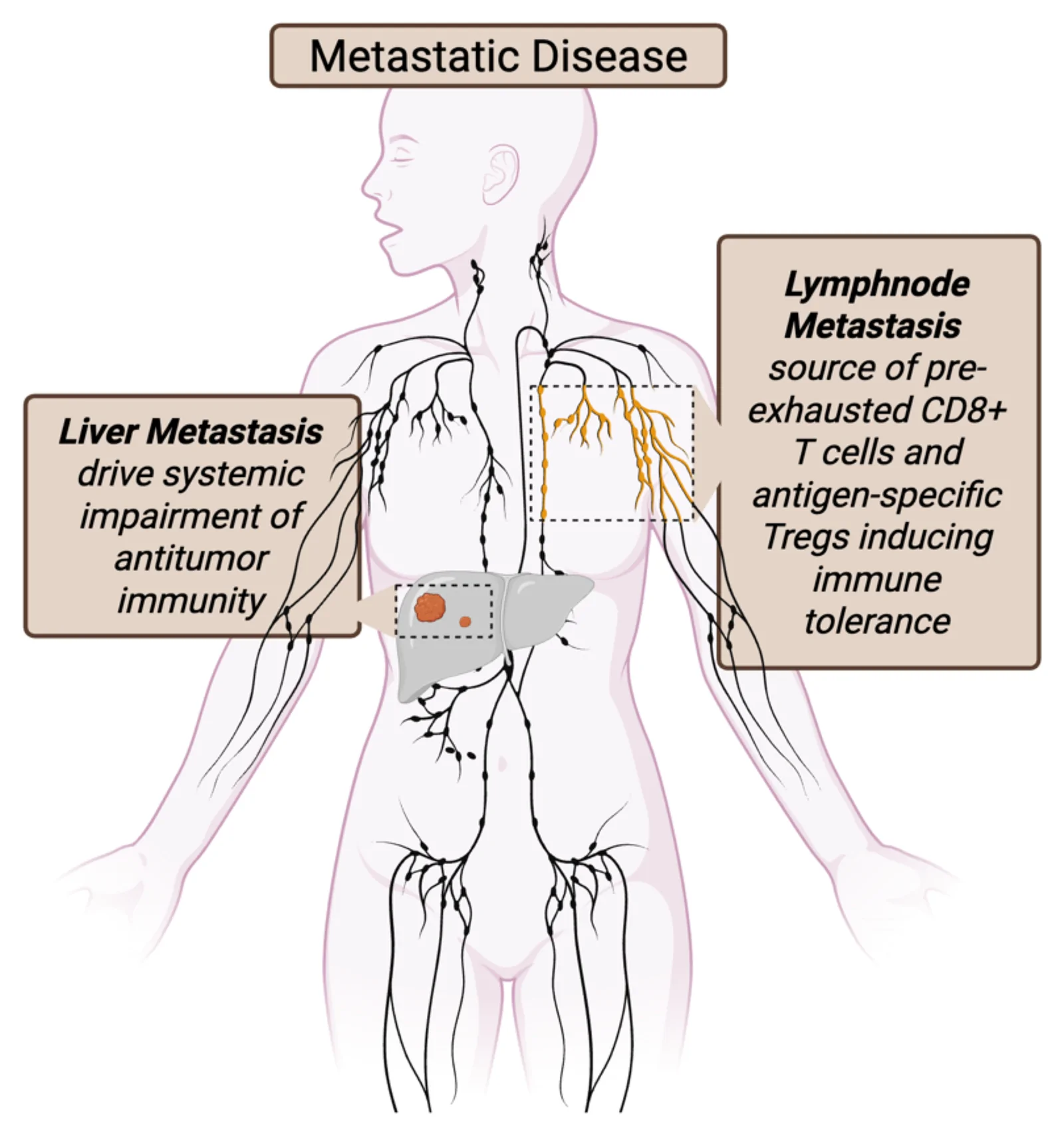
Schematic representation of how metastasis in the liver and lymph nodes induce additional systemic alterations in the immune system influencing cancer immunity.

Cancer progression often culminates in metastasis growing in different tissues. Indeed, metastatic growth is the major cause of cancer patients’ mortality and is treated with systemic therapies such as chemotherapy, radiotherapy and immune-therapy^349^. The metastatic process selects for more aggressive and therapy resistant cells, therefore metastatic recurrences are very hard to treat, which is evident by the fact that stage IV diseases show a survival rate of only between 5% and 30%^350,351^. Contributing to this phenomenon is also the systemic impact of metastasis on the cancer to body connection (Figure 9). The specific organ affected by metastasis plays a pivotal role in determining these systemic effects. For instance, liver metastases create an immunosuppressive environment inducing systemic impairment of antitumor immunity, also due to the reduced number of circulating cytotoxic T cells from the circulation^352^. Consequently, response to immune checkpoint inhibitors is also impaired^353^.

Lymph nodes (LN) are a common site where cancer cells disseminate. The presence of cancer cells in LN is the most informative prognostic factor for most solid tumours^354^. However, the mechanisms by which LN metastasis influences tumour progression are unclear. Since the LN is a critical site where immune cells are educated, we could expect a systemic increase in anti-tumour antigen specific cytotoxic T cells. Yet, data from head and neck squamous cell carcinomas show that non-metastatic tumour-draining LNs are a source of pre-exhausted CD8+ T cells, which migrate to the tumour, pointing at a critical role of LN in anti-tumour immune responses^355^. Moreover, metastasis growing in LNs were reported to induce antigen-specific Tregs that promote further distant metastasis, in line with the generation of tumour-specific immune tolerance^356^.

These systemic immune alterations driven by metastasis growth may also contribute to the development of cancer *cachexia*, a severe, multifactorial syndrome characterised by wasting of body mass and a leading cause of cancer-related mortality. Cachexia likely originates from the metabolic cross-talk at play during cancer to body interactions^205^, which culminates in the self-amplification of the host physiological energy conservation mechanisms^357^. The onset of cachexia decreases the bodies resilience to therapies, immunosuppression and can itself cause death. This condition is part of the health deterioration that reduces life expectancy in patients with prolonged cancer disease, resulting from a complex cascade of events causing dysfunctions in multiple organ systems, such as the bone marrow disruption in haematopoiesis, causing a general susceptibility to infections. Moreover, dysfunctions in all major organs (brain, heart, pancreas, liver, kidney and intestine) are also reported^332^. Those conditions are likely caused by a persistent cancer to body crosstalk, the early stages of which were discussed earlier, via self-amplified mechanisms which are still poorly understood.

## Part 2. Body to cancer connection: how the body physiology influence cancer

**Figure 10:**
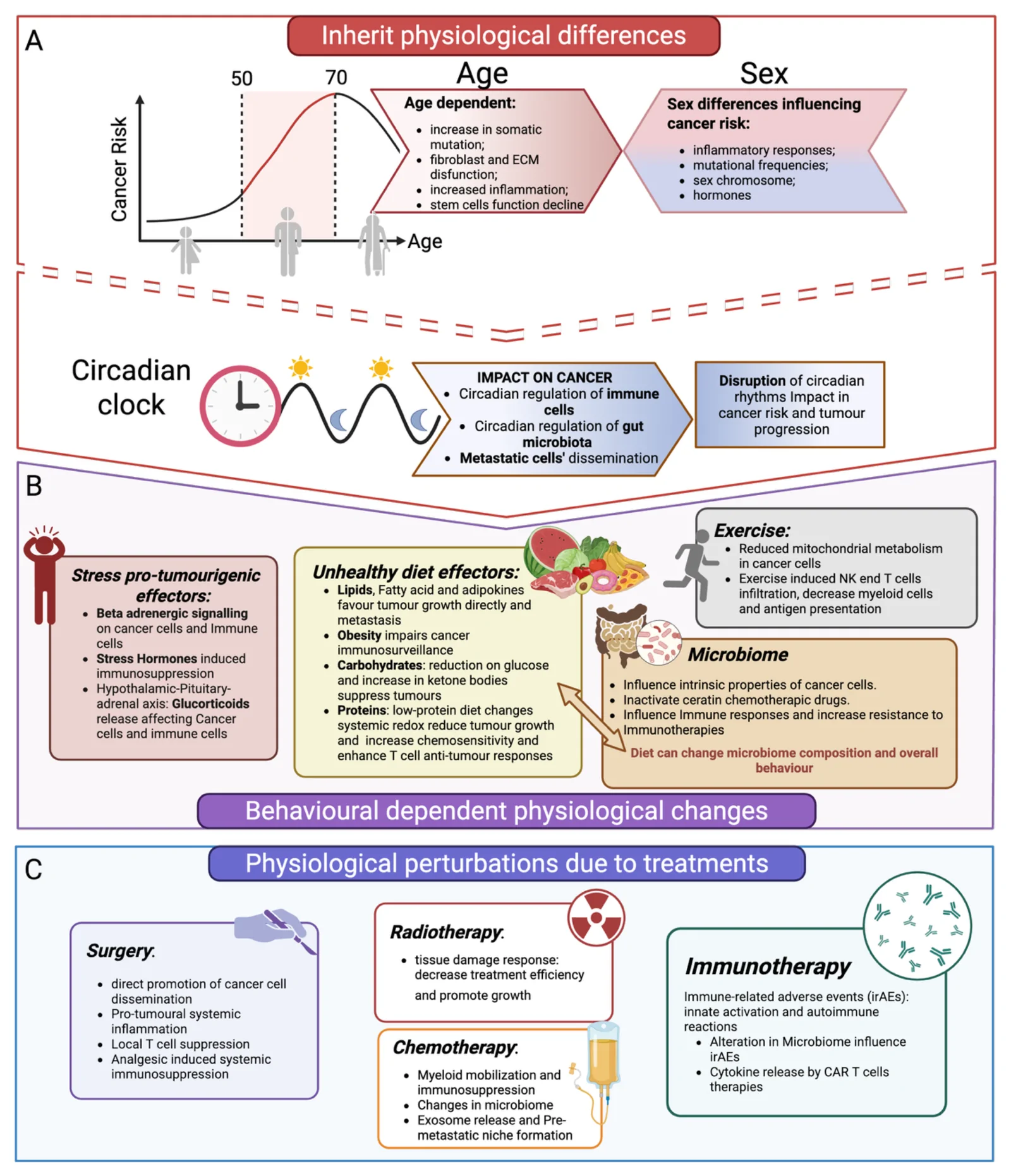
Schematic representation illustrating how distinct pathophysiological states—arising from inherent determinants such as sex, age (A, upper panel), and circadian regulation (A, lower panel), or from extrinsic influences including behavioural factors (B) and anti-cancer treatments (C)—affect cancer progression.

It is long known that exposure to certain environmental factors, which greatly increase the mutagenic burden in an organ, alongside a low-level inflammation, significantly increase the risk of cancer. Smoking is the best-known example where its impact on lung cancer and lung metastasis is mechanistically increasingly well characterized^319,358^. Nonetheless, recently pollution was also reported to play an important role in lung cancer initiation in non-smoking individuals^4^. In the previous section, we discussed alterations in inflammatory cells and platelets, which highlight the potential impact of cancer on cardiovascular conditions. Indeed, the field of cardio-oncology initially developed based on evidence of cardiac dysfunction in patients caused by cancer therapies, a concept termed forward cardio-oncology, in which treatments induce cardiovascular toxicity. However, driven by the strong correlation between these two disease entities within the same individual, the field is now rapidly evolving into a reverse cardio-oncology based on the evidence that the presence of cardiovascular conditions can influence tumour growth or increase the risk of its development (for a more detailed discussion see Meijers et al., 2026^359^). Moreover, as we mentioned earlier another important consequence of cancer systemic manipulation in platelet behaviour is its potential role in predisposing patients to thromboembolism^331^ and this concept could expand to other more complex conditions. This highlights the profound connection between whole-body physiological states and cancer, calling for an in-depth understanding of how host-level alterations shape tumorigenic programs. In the next section, we will analyse the many contexts that can influence cancer through complex physiological mechanisms. Some are inherited, such as sex, age and circadian regulation, or otherwise inevitable, such as surgery, while others are influenced by personal behaviour, including diet, exercise, and stress. (Figure 10).

### 2.1 Inherent determinants

#### Ageing and sex

In the absence of predisposing germline mutations causing cancer in young age, cancer is considered an ***age-related disease*** with over 40% of incidence ranging between 50 and 70 years of age^360^. The traditional explanation for this connection is that cancer originates from oncogenic driving somatic mutations, the frequency of which increases with age. However, recent evidence of oncogenic mutant clones expanding with an age-dependent increased frequency in healthy tissues, have complicated this simple explanation by indicating that mutations alone are not sufficient to cause cancer^2,361^. Nonetheless, in reproductive organs, where genetic determinants of ovarian ageing determine normal variation in the length of fertility, the genetic susceptibility to premature ovarian ageing increases the risk of cancers, suggest a direct connection between tissue ageing and cancer^362,363^. Indeed, the many hallmarks of ageing at the cellular and physiological level overlap with cancer hallmarks^361^. In line with this, many studies indicate that an aged tissue has an increased ability to support the growth of cancer. For instance, physiologic changes due to metabolic alterations play an important role, such as the ageing dependent increase in methylmalonic acid (MMA), which was reported to function as a mediator of tumour progression^364^. Also, local changes in the stromal composition were reported to foster metastatic initiation of melanoma and breast cancer cells in the lung environment^365,366^. Moreover, changes in the progenitor pools of ageing tissue were linked to cancer susceptibility in breast cancer. A study in aged rat mammary glands showed the emergence of progenitor pool, a change linked to expression of midkine (Mdk), a protein that is highly expressed during embryogenesis that plays a key role in tissue repair. Indeed, higher Mdk levels in younger women correlate with increased cancer risk^367^.

However, some of the hallmarks of ageing are antagonistic with cancer, for instance the high stemness activity driving cancer, which is also crucially declining with age^361,368^. Indeed, while, as mentioned above, an aged tissue supports cancer growth and its aggressive behaviour, a recent study in lung cancer demonstrated the ability of aged stem/progenitor cells to respond to an oncogenic mutation with cancer initiation is strongly reduced^369^. This was linked to iron insufficiency in the aged lung stem cell pool.

Given the importance of inflammatory responses for cancer initiation and progression, how immune cells alter their behaviour as a consequence of age-dependent tissue changes must be considered^370^. Moreover, the emergence of clonal haematopoiesis (CH) caused by the onset and expansion of mutations in the hematopoietic compartment of the bone marrow is known to cause age-related dysregulation in the immune system^371^. Besides an increasing predisposition for leukaemia, whole-genome sequencing showed a correlation between CH and lung cancer risk^372^. Key myeloid components in the systemic responses to cancer such as neutrophils harbouring a mutation in the TET2 gene, one of the most common mutation in CH, altered their function and showed increased inflammatory responses^373^. In line with this, the presence of TET-mutant myeloid cells intratumorally was recently shown to promote cancer growth in mice and correlated with higher risk of death of lung cancer patients^374^.

In addition to ageing, another important parameter leading to differences in inflammatory responses is ***sex***^375^. For instance, mechanistic studies have shown that immune responses determine sex-specific outcomes in lung and brain tumours^376,377^. Sex differences can also be detected at the level of cancer drivers, suggesting a sex-dependent mutational process^378^. Clinical evidence also suggest that the immune selection might differ in people with different sex and age, which of course could have implication for immunotherapy responses^379^. Few studies indicate that at least some of the sex dependent changes relate to the presence of the sex chromosomes^380^. In the case of Y chromosome, important events were described to be either as a consequence of its loss^381,382^ or by the upregulation of Y chromosome genes^383^. Certainly, sex hormones have a profound effect on body physiology^384^ and can also influence how the organism responds to dietary interventions impacting on longevity^385^ and will therefore have an profound effect on cancer. Indeed, in lung cancer, sex was a crucial factor influencing therapy responses^386^. But differences are emerging not only in the efficiency of cancer therapies, but, and importantly, in the side effects of those treatments, with female patients showing increase drug toxicity compared to males^387^. This calls for an increased awareness of sex-specific effects at all stages of anti-cancer drug development.

Finally, the fact that ageing and sex are strongly overlapping in their influence on cancer must be considered. For instance, neutrophil biology in ageing differs accordingly to sex^388^. This can reflect in sex specific age-dependent differences, such as the age-dependent shift in the lung microenvironment observed in melanoma, with higher IL-17-expressing γδT cells (γδ17) increasing the presence of immunosuppressive neutrophils and leading to increased metastasis specifically in male mice. Another example is the earlier senescence in response to ZNRF3 loss observed in aged adrenal cancer of males leading to a greater immune response and reduced cancer onset. This inflammatory response was reduced in aged females where metastatic progression was increased^389^.

In conclusion, physiological differences due to sex have a significant impact on cancer development and therapy response, therefore this is an important parameter to consider when using suitable models to study cancer as well as in clinical studies.

#### Circadian rhythm

Increasing evidence indicates that disruption of circadian rhythms, the cell-autonomous daily oscillations that governs vital endocrine, metabolic and immune functions, can influence cancer initiation and progression. Large epidemiological studies have linked night shift work and chronic jet lag to increased cancer risk, particularly for breast cancer^390^. Compelling evidence from animal models supports this, with genetic disruption of core clock components or environmental perturbations (such as irregular light cues) accelerating tumour initiation and progression across multiple cancer types (reviewed in Fortin et al 2025^391^). Mechanisms include deregulation of oncogenic signalling, metabolic dysfunction, immune modulation and impaired cell cycle control and DNA repair. Conversely, studies in glioblastoma and acute myeloid leukaemia demonstrate a dependency on core clock transcription factors BMAL1 and CLOCK for cancer stem cell maintenance^392,393^, indicating a cancer/context-specific role for circadian machinery in tumorigenesis. Metastatic dissemination may also be subject to circadian regulation, with breast cancer patients and murine models exhibiting a surge in metastatic-proficient CTC release during sleep, under the influence of circadian-regulated hormones^394^.

Circadian disruption also modulates tumour growth and metastatic potential by reshaping local and systemic immune landscapes. Chronic jet lag in murine models of melanoma and breast cancer drives an immunosuppressive shift in the tumour immune microenvironment via alterations in chemokine/cytokine regulatory networks, promoting cancer cell proliferation, dissemination and metastasis^395,396^. Circadian-regulated chemokine expression is also evident in glioblastoma, with CLOCK-induced OLFML3 secretion driving tumour infiltration of immunosuppressive microglia^397^. Systemically, circadian-regulated trafficking of neutrophils into naïve lungs establishes a diurnal transcriptional program that shapes the lung’s susceptibility to melanoma cell seeding, suggesting pre-metastatic niche formation is also under circadian influence^398^. Tumour-infiltrating lymphocytes, particularly CD8+ T cells, similarly exhibit circadian oscillations in both frequency and phenotype. Preclinical studies attribute this to diurnal T cell trafficking through tumour vasculature^399^, circadian regulation of PD-L1 expression on myeloid cells^400^ and rhythmicity in dendritic cell lymph node trafficking and co-stimulatory molecule expression^401^. Notably, these studies demonstrate enhanced efficacy of CAR T cell therapy and checkpoint blockade when coordinated with circadian T cell activity. This aligns with retrospective clinical analyses demonstrating improved ICI responses with morning administration across multiple cancer types^402^. This concept of circadian-aligned therapeutic delivery, or chronotherapy, is not unique to immunotherapy, with morning administration of chemotherapy and radiotherapy long been associated with reduced toxicity and enhanced responses across a range of cancers^403^. Finally, circadian regulation also shapes host-microbiome interactions, with gut microbiota displaying robust circadian rhythmicity and its disruption associated with colorectal cancer progression via dysbiosis and intestinal barrier defects^404^, and likely other cancers through systemic metabolic and immune perturbations.

### 2.2 Extrinsic and behavioural driven challenges

#### 2.2.1 Stress

The role of stress in cancer development was first implicated in ancient Greece, when the physician Galen associated higher incidence of tumours of the reproductive organs with women with ‘melancholic natures’^405^. However, it remains challenging to further substantiate this longstanding myth by the equivocal epidemiology findings in the past few decades^405–407^. Considering the extensive confounding factors in human studies and other more extensive reviews available^405,406^, here we will focus on the causal evidence between stress and cancer shown in experimental animal models.

##### Experimental models of stress

Both physiological and psychological factors could induce stress in humans. Acute stress, which lasts minutes to hours, could be adaptive and trigger ‘fight or flight’ responses; on the other hand, chronic stress, which lasts days or longer, could lead to pathological maladaptations^408^, including dysregulation of neuroendocrine systems and immunosuppression. Numerous stress conditions have been introduced in animal studies, mainly classified into three major categories: physical, psychosocial, or a mixture of both^409^. Physical stressors include repetitive injection, laparotomy, foot-shock, etc, while social stressors include social isolation, social defeat^410^, and others^411^. The stressors could also be either acute or chronic; predictable or unpredictable. Notably, different stressors have been reported to lead to distinct behaviour outcomes and biological alterations^409^. In cancer research, chronic restraint is one of the most commonly used methods for chronic stress induction^140,412–414^, although other methods have also been employed^406^. Interestingly, it has also been reported that room-temperature housing condition induces chronic cold stress in mice, while thermoneutral ambient temperature (30–31 °C) significantly suppresses tumour progression^415^.

##### Body response to stress: Activation of both HPA-axis and the SNS

There are multiple ways by which stress alters our physiological status. The stress response is coordinated by the CNS through stimulating the hypothalamic-pituitary-adrenal (HPA) axis^416^ and the sympathetic nervous system (SNS)^186^, two of the most studied neuroendocrine mechanisms by which stress impacts cancer progression^405^. Under stress conditions, the hypothalamus secretes corticotropin-releasing hormone (CRH) to stimulate the pituitary gland, which in turn synthesises and secretes adrenocorticotropic hormone (ACTH). In parallel, the locus caeruleus and other brainstem nuclei release norepinephrine (NE, also known as noradrenaline) and activate the SNS. The two systems converge on the adrenal gland: the ACTH (HPA axis) triggers secretion and release of glucocorticoids (e.g., cortisol or corticosterone) from the adrenal cortex, while the direct innervation by the SNS triggers generation and release of catecholamines (NE and epinephrine) from the adrenal medulla. In addition, the SNS regulates the biological functions in different organ systems via direct innervation^186^, including various lymphoid organs^417^, to regulate both local and systemic immune reactions. For example, intravital imaging shows that lymphocyte migration within the lymph nodes is halted upon acute catecholamine application, suggesting that stress and resultant SNS activation directly contribute to impaired immune response^418^.

##### Stress and tumour initiation

Chronic catecholamine stimulation activates β2-adrenoreceptors, thereby increasing DNA damage and p53 degradation^419^; consistently, chronic stress promotes tumorigenesis induced by ionizing radiation in p53 heterozygous mice^412^.

##### Stress and tumour progression

Stress has been shown to promote cancer progression in various animal models, including GEMMs of PDAC^140^ and an orthotopic xenograft model of ovarian carcinoma^413^, through activating the β adrenergic signalling pathway. Chronic restraint also promotes lung metastases in both GEMM and transplant MMTV-PyMT models of breast cancer, as well as splenic metastases in an orthotopic PDAC model^420^.

###### Impact on cancer cells

Chronic stress increases circulating levels of glucocorticoid, which acts via its receptor to exert direct pro-metastatic effects on cancer cells. Stress hormone levels are higher in patients with metastatic breast cancer^421^, and glucocorticoid receptor signalling directly promotes metastasis and decreased survival in transplantation models of breast cancer^422,423^. Adrenergic receptors are also expressed by various cancers^140^, and respond to increased adrenergic signals to promote cancer progression.

###### Impact on the TME and the pre-metastatic niche

The SNS plays a crucial role in regulating immune cell functions via adrenergic signalling^424^, which directly promotes T cell exhaustion in PDAC^146^ and leads to heightened metastasis in breast cancer via recruitment of stress-induced macrophages into the primary tumour^425^. Glucocorticoid also modulates the pre-metastatic niche, which includes recruiting tumour-associated macrophages^426^ and promoting the formation of neutrophil extracellular traps^420^, thereby altering the lung microenvironment to promote lung metastasis. Stress-induced glucocorticoid secretion also acts on dendritic cells to cause immunosuppression and treatment resistance^410^. In addition, stress stimulates angiogenesis in an orthotopic model of ovarian cancer^413^, and promotes lymphatic vasculature remodelling in an orthotopic model of breast cancer, resulting in aggravated metastasis^414^.

Finally, although studies on how circulating stress hormones promote the progression of various cancer types have been fruitful and yielded convincing results, it’s worth noting that exercise also increases circulating catecholamine levels. However, paradoxically, exercise-induced catecholamines have been shown to suppress cancer progression in animal models via β adrenergic signalling, both directly on cancer cells^427^ or indirectly through immune cells^428^. Therefore, in the future, in addition to focusing on circulating factors, local release of catecholamines^429^ and different neural circuits activated in stress response and in exercise should be identified and carefully compared, to allow detailed mechanistic studies of their impact on cancer.

#### 2.2.2 Diet and exercise Diet

Dietary composition shapes the nutrient supply and metabolite species present in the circulation and tumour interstitial fluid^430^, profoundly influencing tumour development, progression and treatment response. Dietary modifications represent a promising strategy for reducing tumour growth through limiting nutrient availability within the TME, exploiting tumour-specific metabolic vulnerabilities or augmenting efficacy of anti-cancer therapies.

##### Lipids

Obesity, defined by a high body mass index (BMI) (≥30 kg/m^2^) and largely arising from excess dietary fat intake, is strongly associated with increased cancer incidence and mortality across many solid tumours including breast, endometrial and colorectal cancers. Mechanistically, obesity-associated systemic elevation of growth hormones, insulin, glucose, fatty acids and adipokines such as leptin can directly promote cell proliferation and survival, favouring tumour growth (reviewed^431^). For instance, the uptake of fatty acids by cancer cells, a process amplified under obese conditions, serves as a direct metabolic substrate to fuel tumour growth and metastasis^432–435^. Reducing tumour access to unsaturated fatty acids via dietary interventions impairs tumour growth in murine *KRAS*-mutant PDAC^436^.

Recent studies using clinical samples and murine tumour models have shown diet-induced obesity may indirectly contribute to tumorigenesis via immune dysregulation and impaired cancer immunosurveillance. Tumour progression in obese tumour-bearing mice has been linked to CD8^+^ T cell effector dysfunction and PD-1-driven T cell exhaustion, mediated at least in part by increased circulating levels of leptin^437–439^. These studies also demonstrated reduced CD8^+^ T cell infiltration and activation in tumour tissues from patients with obesity compared to non-obese patients. Using a multi-omics approach, Ringel and colleagues linked impaired CD8^+^ T cell infiltration and function to obesity-driven metabolic reprogramming, with enhanced fat utilisation by cancer cells paradoxically leading to a fatty-acid depleted TME in obese mice that compromised anti-tumour immunity^16^. Impaired effector T cell responses were also recently found in the TME of obese melanoma-bearing mice, which was reversible with diet-induced weight loss but not with the obesity-combatting medication semaglutide^440^. Importantly, despite compromised tumour immunity, obesity is associated with enhanced efficacy of checkpoint blockade and improved survival in both tumour-bearing mice and cancer patients (the ‘obesity paradox’)^437,439,441^. Beyond T cells, reduced cancer immunosurveillance has been linked to obesity-driven dysregulation of innate immunity, with obesity selectively inducing PD-1 expression on TAMs which then dampens macrophage functions including phagocytosis and T cell stimulatory potential^442^. Modelling variable human dietary habits via cyclical HFD triggers striking IL-1β-dependent reprogramming of neutrophil progenitors, although the consequence in the context of cancer was not examined^443^. Both murine models and patients with obesity show impaired NK cell-mediated anti-tumour responses linked to the induction of peroxisome proliferator-activated receptor (PPAR)-mediated lipid accumulation^444^. The resulting ‘metabolic paralysis’ blunts NK cell function by inhibiting the production and intracellular trafficking of cytotoxic machinery.

Beyond the primary tumour, a high fat intake can prime distant organs for subsequent metastatic colonisation, reminiscent of pre-metastatic niche formation. Mice exposed to a HFD show increased fatty acid availability in the liver and lungs that promotes subsequent metastasis^204^, while diet-induced obesity triggers lung neutrophilia that supports DTC extravasation and outgrowth^15,445^. Dietary fatty acids, and specifically palmitic acid, can endow cancer cells expressing fatty acid receptor CD36 with enhanced metastatic potential via CD36-dependent lipid metabolism^435^, with this high metastatic capacity preserved long-term via crosstalk with tumour-associated Schwann cells^446^.

##### Carbohydrates

High-carbohydrate diets, such as excess consumption of dietary glucose and fructose, are linked to enhanced tumorigenesis and immune evasion^447,448^. The fasting-mimicking diet, in which subjects receive periodic cycles of calorie-restricted, low-carbohydrate low-protein diets that lowers blood glucose, insulin and IGF-1, has shown potential as an adjunct to anti-cancer therapy. Enhanced efficacy has been observed in cancer patients or tumour-bearing mice receiving fasting-mimicking diet in conjunction with cytotoxic chemotherapy^449,450^, endocrine therapy^451^ and immunotherapy^452,453^. Consistent with the latter, Vernieri and colleagues found a potent remodelling of systemic and intratumoral immunity in cancer patients receiving standard of care therapy in combination with fasting-mimicking diet^454^.

An alternate dietary intervention for carbohydrate reduction is the ketogenic diet, a high-fat very low carbohydrate diet that reduces blood glucose while concomitantly increasing circulating ketone bodies. There is emerging evidence for a tumour-inhibitory role of ketone bodies, in particular β-hydroxybutyrate, which potently supresses tumorigenesis through direct effects on cellular proliferation^455^ and exhibits an immunomodulatory function^456,457^. Oral administration of β-hydroxybutyrate synergises with immune checkpoint inhibitors (ICI) in multiple orthotopic murine tumour models^457^, including an immunotherapy-refractory prostate cancer murine model^458^. Enhanced effector and regulatory T cell immune capacity and memory T cell formation has also been observed in Peripheral Blood Mononuclear Cells (PBMCs) exposed to β-hydroxybutyrate *in vitro* and in healthy volunteers on a short-term ketogenic diet^459^.

##### Protein

Altering amino acid availability in the TME via low-protein diet or depletion of specific amino acids has shown promising anticancer efficacy in preclinical models^460,461^. Methionine restriction elicits changes in systemic redox and nucleotide metabolism in both humans and tumour-bearing mice, creating a metabolic vulnerability that enhances chemosensitivity in xenograft models of colorectal cancer^462^. Dietary restriction of serine and glycine improves survival in some murine tumour models, although tumour reliance on exogenous serine depends on the oncogenic landscape and tissue of origin^463^. A potent reduction in tumour growth^464^ and metastatic burden^465^ was observed following restriction of dietary arginine and asparagine, respectively, conversely glutamine supplementation elicits anti-cancer activity and synergises with BRAF inhibition in melanoma^466^ (see Tajan et al^460^ for additional examples). Beyond cancer cell metabolism, protein availability also influences host immune functions. A low-protein diet enhances T cell anti-tumour responses in across multiple murine tumour models^467^, while low protein or specifically methionine and cysteine restriction can trigger phenotype switching in TAMs to enhance tumoricidal capacity^468^.

#### Exercise

Strong epidemiological evidence suggests regular physical activity decreases cancer incidence across multiple tumour types^469^ and lowers risk of recurrence and mortality in breast, prostate and colorectal cancer^470^. Exercise triggers widespread changes to body physiology through the systemic release of hormones, cytokines and growth factors from various tissues and organs including skeletal muscle, bone and adipose tissue. Recent multi-omic profiling of the molecular alterations induced by endurance training comprehensively mapped the whole-body physiological response to exercise, including changes to immune and metabolic pathways^471^. In the context of cancer, this systemic response drives alterations to cellular metabolism, immune regulation and vascular remodelling in the TME, hindering tumour growth and progression^472,473^. As an example, the anti-tumorigenic effects of exercise were linked to reduced mitochondrial metabolism and respiratory capacity in tumour cells from exercised mice compared to sedentary animals in colorectal PDX xenografts^474^ and a murine mammary tumour model^475^.

Preclinical studies in tumour-bearing mice and emerging clinical data supports the exercise-dependent modulation of innate and adaptive immunity (recently reviewed^476^). Exposure of mice to regular exercise (voluntary wheel running) significantly reduced tumour growth and lung metastasis in mice inoculated with B16 melanoma cells, attributed to epinephrine-mediated systemic mobilisation of NK cells followed by IL6-induced tumour homing^477^. Explorative analyses in cancer patients show a trend towards exercise-induced NK infiltration and activation^478,479^. Preclinical studies in murine models of PDAC^480^, melanoma^481^ and breast cancer^482–484^ have linked exercise-mediated reduction in tumour growth to increased infiltration and effector function of CD8+ cytotoxic T cells, perhaps mediated by IL-15 signalling^480^. Higher tumour CD8+ T cell infiltrates and expression of granzyme B were similarly observed in PDAC patients who participated in a preoperative exercise training program^480^. Exercise may also augment anti-tumour immunity through TAM polarisation and enhanced DC antigen presenting capacity^481^ or reduction in tumour-infiltrating myeloid cells^480,485,486^.

A growing body of evidence suggests exercise-induced alterations in the TME may improve therapeutic efficacy. Exercise-induced vascular remodelling enhances chemotherapeutic drug delivery and efficacy in murine and human PDAC^487,488^ and syngeneic mammary tumour mouse models^489^. Consistent with its immunomodulatory function, exercise sensitizes tumours to anti-PD1 immunotherapy in preclinical models^480,483,485,490^. Clinical studies are needed to confirm whether the exercise-induced mobilisation and priming of effector immune cells can be harnessed to boost immunotherapy responses (reviewed^491^).

### 2.2.3 Microbiome and Cancer

Analysis of faecal samples and other mucosal biospecimens has revealed differences in the richness and diversity of microbiota communities in cancer patients in comparison to healthy individuals^492,493^. Gut dysbiosis, characterised by an unbalanced composition of commensal species in mucosa sites, is associated with increased cancer risk, underscoring the role of microbial equilibrium in oncogenic resistance. Intestinal microbiota has been shown to influence cancer fate through direct interactions with malignant cells or indirectly by shaping anti-tumour immune responses^492,493^. Conversely, the prevalence and abundance of certain gut commensals correlates with increased immunity to cancer and enhanced efficacy of immunotherapy^492,493^. Similarly, in various patient cohorts, antibiotic treatment correlates with poor responses to immunotherapy, highlighting the beneficial role of individual species to favourable responses to anti-cancer therapy^494^. Although the mechanisms by which microbiota inhibits or promotes cancer are not yet fully understood, they are considered critical factors in both cancer onset and therapeutic outcomes.

#### Local interactions

Disruption of the intestinal microbiota increases susceptibility to colonisation by opportunistic pathogens or the uncontrolled expansion of certain commensal species, both of which can exhibit oncogenic actions. Indeed, certain bacterial species, including *Helicobacter pylori, Fusobacterium nucleatum*, have been implicated in the development of gastric and colon cancers^495,496^. These microbes can contribute to carcinogenesis through the production of virulence factors that disrupt the tissue barrier, foster chronic inflammation, and enhance tumour progression by promoting immune evasion and boosting metastatic potential^497^.

Microbiota dysbiosis extend beyond gastrointestinal malignancies. Also, outside the gut, tumours harbour unique microbial communities that might influence cancer progression and treatment responses. Advanced sequencing technologies revealed the presence of intratumoral bacteria and fungi across various malignancies, challenging previous notions that tumours are sterile environments^498,499^. Malignant transformation and adverse reactions of anti-cancer therapies can impair the barrier integrity of mucosal tissues potentially leading to translocation of commensal species in the tumour bed. Intratumoral microbes have been suggested to shape primary and metastatic cancer both positively and negatively potentially through diverse mechanisms including transformation of the inflammatory milieu of tumours and remodelling of the phenotypic plasticity of cancer cells^500–502^. In a mouse breast cancer model, intracellular bacteria were found to promote lung metastasis by remodelling the actin-cytoskeleton of cancer cells^503^. In a human pancreatic cancer cohort, the presence of a subset of intratumoral bacteria were predictive of clinical outcome and were found to be associated with pathway alterations in cancer cells and distinctive immune signatures^501^. Similarly, T cells with reactivity to microbial antigens have been identified in human melanoma tumours but their contribution to anti-tumour immunity remains unclear^504^. Furthermore, certain tumour-resident microbes can reduce therapeutic outcome by contributing to metabolic inactivation of chemotherapeutic agents or promoting resistance to therapy by altering cancer cell metabolism^505^. The type of microbial species identified in human and mouse cancer predominantly depend on the anatomical area that favours the colonisation, expansion and maintenance of specific phyla. Although, still not fully understood, the contexture of the tumour microbiota may also be influenced by intrinsic properties of the cancer itself^499^. Collectively, despite the increasing evidence of tumour-resident microbiota, its scarcity in the TME poses a major challenge in confidently detecting the microbial signals. Several workflows and computational pipelines complemented with strategies of *in situ* visualisation and *ex vivo* cultures of intratumoral microbial species have recently been implemented to overcome the technical challenges, allowing investigators to confidently define tumour-resident microbes and explore their functional consequence in cancer^498,501,503^.

#### Distal interactions

Despite its local impact on cancer, the gut microbiota can also impact cancers located distally to the gastrointestinal tract. Several studies in mice and humans have identified intestinal commensal species to influence immune responses to extraintestinal cancers and modulate the efficacy of T cell-based immunotherapies^492,493^. Certain gut commensals including *Akkermansia muciniphila* and *Bifidobacterium* were shown to enhance antigen presentation and stimulate cytotoxic T cell responses, while *Clostridium* and *Enterocloster* species reduce immunotherapy efficacy by inhibiting the infiltration of natural killer T (NKT) cells and increasing the accumulation of immunosuppressive T cells in the TME, respectively^506–508^. Probiotics and faecal transplantation from immunotherapy-responders are now tested in phase 1 clinical trials in efforts to explore the therapeutic benefit of manipulating the microbiome to overcome immunotherapy resistance in non-responders^509–511^.

Gut commensals could influence immune responses to peripheral cancers through the release of soluble factors into the blood, including cell wall-associated molecules, metabolites and membrane vesicles enriched in various pattern recognition receptor (PRR) ligands. Short-chain fatty acids (SCFAs) and the purine metabolite inosine enhance T cell function and regulatory pathways essential for anti-tumour immunity^512,513^. Additionally, microbial-derived bile acids can exert tumour promoting action by influencing the immune repertoire in the tumour bed^514^. PRR signalling by microbiota-associated molecular patterns has been shown to enhance type 1 interferon production by monocytes, resulting in dendritic cell accumulation in the TME and cancer control^515^. Furthermore, PRR signalling by microbiota-derived ligands might also contribute to cancer immunity by enhancing ability of dendritic cells to prime naïve T cell responses against foreign antigens^516^. Dysbiosis-induced inflammation can elevate circulating pro-inflammatory cytokines, affecting distant organs and potentially fostering tumour development^517^. In addition to microbial products and associated inflammatory mediators, microbiota-reactive T cells can dictate anti-cancer T cell clonality and invariably shape cancer immunity. Studies have shown that effector T cells generated against microbes can display anti-tumour activity as a result of cross-reactivity between microbial antigens and structurally homologous tumour neoantigens^518^. Previously, microbiota-educated CD8^+^ T cells were found to exhibit qualitatively different immune responses to transplantable tumours in comparison to peripheral CD8^+^ T cells, as measured by increased expression of interferon-stimulated genes and enhanced mitochondrial activity^519^. Furthermore, gut resident CD4^+^ T cells that were originally primed against microbiota antigens in gut-associated lymphoid tissues were found to migrate to tumour-draining lymph nodes and dampen cancer immunity following downregulation of the gut homing receptor MadCAM-1^508^.

#### Diet dictates microbiome-host interactions in cancer

Early-life microbiota is to a certain extent inherited and is influenced by geographical distribution and host genetics. During infancy the mother’s microbes are transferred and colonize multiple organs accounting for more than half of taxa diversity of early-life microbiota in infant’s body^520^. Microbiota matures during ageing, and it can be further shaped by environmental factors. Interestingly, older microbiomes in mice were shown to prevail younger ones in immunotherapy efficacy due to aged-enriched enterotypes that potentiate anti-tumour T cell responses^521^. Dietary habits significantly influence the composition and function of the gut microbiome, thereby impacting cancer risk and progression^522^. Western diets, characterized by high fat and low fibre intake, promote dysbiosis, increasing pro-inflammatory bacterial populations while reducing beneficial commensals^522^. Dysbiotic microbiomes associated with Western diets have been linked to inflammation and implicated in colorectal carcinogenesis^514,523,524^. Conversely, diets rich in fibre, polyphenols, and fermented foods enhance microbial diversity, fostering protective metabolites beneficial for immune regulation and cancer prevention^522^. Indeed, high fibre diet has been shown to enhance gut barrier integrity, support cancer immune surveillance and augment immunotherapy response^515,525^. The ingredient complexity of diets makes it difficult to deconstruct how nutritional interventions control microbiome-dependent immunity to cancer. Recently, it has been shown that a single micronutrient, vitamin D, acts on vitamin D receptor (VDR) of the gut epithelium to enable the gut microbiome to induce potent T cell-mediated immunity, dictating immunotherapy success in pre-clinical models^526^. Other dietary components, including inulin and tryptophan have been shown to improve responses to anti-cancer therapy through direct interaction with the immune system^527–529^. Collectively, dietary interventions aimed at modulating the microbiome present promising strategies for cancer prevention and treatment enhancement. Further research into diet-microbiome interactions will be instrumental in developing personalized nutritional strategies for cancer patients.

## 2.3 The consequence of anti-cancer therapies

### 2.3.1 Surgery

Surgery is standard of care and a primary treatment option in most cancers. However, the notion that the surgical procedure itself may promote cancer cell dissemination and subsequent metastasis emerged decades ago^530,531^. The envisaged scenario was that the mechanical impact of the surgical procedure on the cancer mass may cause cells shedding from the primary tumour and disseminate to metastatic sites^532^. In line with this hypothesis, the increase of CTCs after surgery has been shown in different pre-clinical models^533^ and clinical cases have even reported cancer cell seeding after small mechanical procedures such as a tumour biopsy^534^.

Apart from a direct, mechanical effect dependent on the surgery, it must also be considered that a surgical trauma, like other tissue traumas, induces both a local and a systemic inflammatory response. This systemic inflammation is likely to influence the survival and possibly the proliferation of cells already disseminated at the time of the surgical intervention. This is supported by a substantial body of both pre-clinical and clinical evidence showing that homeostatic changes due to tissue trauma, as well as wound healing processes associated with surgery, promote micrometastasis growth^535^. It is worth noting that this effect accompanying any surgery, not necessarily related to cancer resection, could also influence cancer progression in patients with an undetected cancer.

Importantly, the mechanisms by which the systemic changes resulting from surgery play a role in accelerating metastasis growth remain unclear. Preclinical studies showed that while breast cancer metastatic growth can be restricted by a tumour specific T-cell response, this is impaired due to a wound-healing response following tumour resection^536^. This may involve a crosstalk between innate and adaptive immunity at the metastatic site^537^.

Indeed, surgical stress response is a recognised still complex phenomenon involving an intricate network of interactions occurring between the immune, the endocrine and the metabolic system. The immediate post-operative response is characterised by the increase in anti-inflammatory agents such as IL-4, IL-10 and TGF-β, promoting a state of immunosuppression^538^. This is only transitory, and while it can last for about two weeks^539,540^ it peaks in the very first days post-surgery^541^. It may therefore be possible to follow the magnitude of the immunosuppression by measuring the levels of different biomarkers in the blood^542–544^. To add to the complexity, other aspects of surgery should also be considered, such as the administration of anaesthetic drugs and opiates during and after the procedure that directly contribute to the observed systemic immunosuppression^545^.

It is important to highlight that while a general immunosuppressive state associated with mechanisms of wound healing early after surgery could provide an opportunity for disseminated cancer cells to outgrow, the absence of a primary tumour and its associated effects can ultimately restore effective adaptive immune responses^546^. Therefore, since a combination of both detrimental and beneficial effects on the immune system can be associated with the surgical removal of the tumour, more research is needed to understand how these changes may depend on a particular cancer type or the stage of the disease. This could lead to the identification of a window of opportunity during which patients are more susceptible to residual disease growth both at the primary and metastatic sites. While this possibility was already suggested several years ago^531^ this has not translated yet in pharmacological interventions aiming to modulate the immune system to avoid an excessive systemic immunosuppression post-surgery, particularly critical in potential sites of metastasis.

However, potential therapeutic options in the area continue to appear. For example, low-dose rapamycin has immunomodulatory effects and its administration reduced surgery-induced immune dysfunction and in turn re-established immunotherapy efficacy in preclinical models of bladder cancer^547^. Similar therapeutic avenues using more effective adjuvant drugs could emerge from a better understanding of how the inflammatory scenario changes systemically across different tissues due to post-surgery immunosuppression.

### 2.3.2 Systemic Effects of Chemo- and Radiotherapy

Anti-cancer therapy induces a spectrum of systemic changes in the host cell and tissue physiology, encompassing both intended action and unintended reaction to treatment. Accumulating evidence suggests that the therapy-induced physiological response of the host, including systemic release of cytokines and immune mobilisation, may dampen the desired anti-tumour effect of treatment, and, in some cases, even promote tumour repopulation and/or metastasis (reviewed^533^). For example, radiotherapy and some chemotherapies can enhance their efficacy through engagement with the adaptive immune system, in a process termed immunogenic cell death^548^. However, in some contexts, treatment-induced tissue damage can elicit a pro-tumoural inflammatory reaction that may hinder efficacy or promote tumour dissemination. Although majority of studies demonstrating pro-tumoral effects of therapy have utilised preclinical models, supportive evidence from patients with cancer is accumulating. Understanding how these reactive host effects can alter tumour fate following treatment will likely unlock new therapeutic targets or combination approaches to enhance treatment efficacy and durability.

Conventional ***chemotherapy*** has remained the mainstay systemic treatment for many cancers and rapidly induces the death of highly proliferative cells. For several chemotherapeutic agents, such as platinum-based derivatives and anthracyclines, their therapeutic efficacy also has a considerable immunological component through the induction of immunogenic cell death, in which a tumoricidal immune response is mounted in response to DAMPs and neo-epitopes released by dying cancer cells. However, the full potential of the chemotherapy-induced anti-tumour response is often not met due to the involvement of immunosuppressive mechanisms, including the mobilisation and modulation of myeloid cells with immunosuppressive functions such as neutrophils, monocytes and macrophages^549–551^. More recently, the systemic release of B-cell derived EVs was found to blunt chemotherapy efficacy by attenuating CD8+ T cell effector responses^552^. Interestingly, chemotherapy-induced alterations to the gut microbiome can help shape the adaptive immune response^553,554^, indeed clinical studies of cancer patients show receipt of antibiotics during platinum chemotherapy is associated with reduced treatment response and overall survival^555^. Accumulating evidence suggests that systemic host responses triggered by chemotherapy may paradoxically augment cancer cell dissemination and outgrowth in secondary tissues. Adjuvant doxorubicin treatment (but not cisplatin) induces expression of complement factors in lung fibroblasts, shaping an immunosuppressive pre-metastatic niche in the murine lung via myeloid cell recruitment and T cell dysfunction^556^. Paclitaxel treatment can induce lysyl oxidase secretion by CD8^+^ T cells both systemically and in the lungs, which in turn leads to pulmonary ECM remodelling and enhanced metastasis^557^. Both paclitaxel and gemcitabine have also been linked to exacerbated lung metastasis via bone-marrow derived myeloid cell mobilisation, with treatment-induced accumulation of inflammatory monocytes^558^ or CCR2^+^ macrophages^559^ priming lung tissue for incoming cancer cells. In another elegant example of pro-metastatic effects of chemotherapy, neoadjuvant chemotherapy triggered the release of breast tumour-derived EVs enriched with Annexin A6, driving endothelial activation and monocyte expansion in the pre-metastatic lung that enhanced lung metastasis^560^. Annexin A6 can also be detected in circulating EVs of breast cancer patients undergoing neoadjuvant chemotherapy. Tumour-derived small EVs carrying inflammatory glycoprotein PTX3 have also been linked to chemotherapy-induced pro-metastatic lung priming^561^. In the context of established metastases, lung neutrophil recruitment and NET formation in response to chemotherapy can blunt treatment responses in murine breast cancer metastatic models^323^, with the presence of NETs also correlating with chemoresistance in a small cohort of breast cancer patients with advanced disease.

#### Radiation therapy

(RT) is a standard of care treatment modality used to treat most solid tumours. Alongside its genotoxic action, RT can elicit immunomodulatory effects through the release of DAMPs, cytokines, chemokines and tumour neoantigens (reviewed^562^). This can induce both innate and adaptive immune responses, resulting in systemic changes in the host that influence tumour growth, survival and spread. RT-mediated induction of systemic antitumour immunity can, in some cases, enable control of distant metastases, a phenomenon known as the abscopal effect^563^. However, this effect is rarely observed in patients, likely because immune tolerance mechanisms at play during treatment can hamper the development of robust abscopal responses. Seminal case reports and clinical studies from metastatic cancer patients reveal boosting of abscopal response rates through combining RT with immunotherapy^564–566^, for instance abscopal responses and disease control were demonstrated in 30% of patients treated with RT and GM-CSF^565^. The growing consensus is that RT and immunotherapy can synergise to unleash the anti-metastatic power of the adaptive immune system (reviewed^567^). Proposed mechanisms include enhanced tumour immunogenicity through RT-induced cellular stress and liberation of neoantigens, and increased immune mobilisation triggered by damage signals and immunostimulatory cytokines released from the irradiated TME. In murine tumour models and patients treated with RT and immune checkpoint inhibitors, an increase in circulating tumour-derived IFN-β may be essential for T cell priming and the induction of abscopal responses^567,568^. Strong anti-tumour responses have been observed when RT is combined with dual immunotherapy targeting non-redundant immune tolerance mechanisms^569,570^, or when high dose RT and ICI is combined with low-dose radiation to secondary metastases^571,572^.

On the other side of the coin, preclinical studies suggest the beneficial effects of RT may be counteracted in certain instances by the induction of systemic alterations that promote tumour invasion and metastatic spread. RT triggers rapid neutrophil mobilisation to the TME^573^ and NET deposition^574^, with the latter linked to immunosuppression and radioresistance in invasive bladder cancer. Radiation exposure of healthy lung tissue also triggers a strong neutrophilic response, characterised by neutrophil influx, activation and degranulation, priming the organ for metastatic seeding^575^. Interestingly the pro-metastatic activity of neutrophils was mediated by activation of a tissue regeneration response in the lung parenchyma, linking treatment-induced tissue damage to inadvertent tumour progression. Systemic mobilisation of suppressive myeloid cells following RT has also been linked to lung PMN formation and metastasis in oesophageal squamous cell carcinoma, driven by small EVs released from dying tumour cells^576^.

### 2.3.2 Systemic Effects of Immunotherapies

Over the past 15 years, biological therapies targeting the immune system have been incorporated into numerous clinical protocols. While immunotherapies are designed to specifically target the TME, they also induce systemic effects, which may underlie the diverse range of side effects observed in various organ systems, including cardiovascular, dermatological, endocrine, gastrointestinal, neurological, and pulmonary compartments^577^. Immune-related adverse events (irAEs) arise as a result of either autoinflammatory responses, driven by innate immune activation, or autoimmune reactions, characterized by the presence of autoantibodies and antigen-specific memory T cell responses^578^. These irAEs have been most extensively studied in the context of immune checkpoint inhibitors (ICI), where both short-term and long-term irAEs have been reported^579^. Notably, the occurrence of chronic irAEs associated with sustained inflammation or autoimmune-like phenomena may suggest systemic immune remodelling mediated by epigenetic mechanisms.

Importantly, the spectrum and severity of irAEs differ depending on the type of immunotherapy, even within the same category of agents^580,581^. For example, gastrointestinal irAEs are more frequent and severe with *CTLA-4 blockade* compared to *PD-1 or PD-L1 inhibitors*^582^. Emerging evidence links these adverse effects to alterations in the microbiome, with studies suggesting that gut microbial composition may influence irAEs^583^. Interestingly, microbiome modulation has been shown to mitigate gastrointestinal irAEs without compromising therapeutic efficacy^584,585^. This may open innovative therapeutic avenues for combination therapies.

Beyond ICIs, other T cell-engaging therapies, such as bispecific antibodies and *chimeric antigen receptor (CAR) T cell therapies*, have also demonstrated systemic effects, including severe toxicities. Cytokine release syndrome (CRS) is a notable complication, triggered by inflammatory cytokines and chemokines released during CAR T-cell activity^586^. Interestingly, apart from the same CAR T cells administered, myeloid cells, including monocytes and macrophages, have been identified as key contributors to CRS through the production of cytokines such as IL-1 and IL-6^587–591^. These findings underscore the systemic immune changes that can result from targeting specific immune cell subsets, further emphasizing the need to better understand and manage the broader immune rewiring induced by these therapies.

Importantly, while systemic immune activation following immunotherapy may contribute to adverse effects, systemic immunity is also promoting therapeutic efficacy^546^. For instance, systemic dendritic cell activation has been shown to enhance T-cell responses and improve anti-tumour immunity in preclinical models of breast cancer^592^. As immunotherapies aim to modulate the immune response, maintaining a balance between promoting effective anti-tumour immunity while preventing autoimmunity poses a significant challenge^578^. Most currently used immunotherapies target adaptive immune cells. However, a deeper understanding of the dynamic interactions between adaptive and innate immune pathways is essential for improving therapeutic efficacy while minimizing adverse effects^581^. Future research should integrate data from studies measuring serum cytokine signatures post-immunotherapy^593–596^ with insights from molecular studies investigating irAE mechanisms using advanced mouse models^597–599^. These models could also be used to better understand the systemic changes dependent on different immunotherapies in organs that are potential sites of metastasis.

## Concluding Remarks

This review has discussed the complexity of the cancer condition and the multiple dimensions, from local to systemic, at which cancer shapes the body physiology. Understanding this alternative whole-body physiology induced by the carcinogenic program is important not only to manage patients with cancer and the side effects they develop over time, but also to develop better treatment strategies. Moreover, as elaborated in the second part of this review, this understanding is paramount to clarify how other physiological conditions can feed into the cancer disease and contribute to disease outcomes within diverse cancer patient populations. For these reasons studying the complex interplay between cancer and the body should be a priority area when aiming to develop personalised and more effective anti-cancer treatments.

## Clinical Highlights

- Cancer–body connection, the local dimension: because the cancer mass is built through the interaction of cancer cells and cells derived from the local tissue environment, targeted therapies must incorporate and account for the treatment responses of this local environment.
- Cancer–body connection, the systemic dimension: cancer is able to interfere with many aspects of body physiology, impacting disease progression and potentially serving as biomarkers.
- Cancer–body connection, the systemic dimension: the perturbed body physiology in cancer patients should be better studied, as it can influence how patients respond to cancer therapy as well as to other non-cancer-related insults, and may predispose them to additional disease conditions, which complicate treatments.
- Preclinical studies lead the way in capturing cancer–body connections, including at early stages of cancer development when the disease is not yet clinically manifest, but efforts to identify these changes in cancer patients should be encouraged through clinical awareness, from the stage of early diagnosis through disease progression.
- Body to cancer connection: greater investment at both the preclinical and clinical levels is needed to understand how alterations in physiological states impact cancer predisposition and cancer progression; a deeper understanding of this connection represents one of the most powerful ways to reduce global cancer risk.
- Altering whole body physiology represents an opportunity to enhance cancer personalize therapies.
